# Collagen-derived proline promotes pancreatic ductal adenocarcinoma cell survival under nutrient limited conditions

**DOI:** 10.1038/ncomms16031

**Published:** 2017-07-07

**Authors:** Orianne Olivares, Jared R. Mayers, Victoire Gouirand, Margaret E. Torrence, Tristan Gicquel, Laurence Borge, Sophie Lac, Julie Roques, Marie-Noëlle Lavaut, Patrice Berthezène, Marion Rubis, Veronique Secq, Stéphane Garcia, Vincent Moutardier, Dominique Lombardo, Juan Lucio Iovanna, Richard Tomasini, Fabienne Guillaumond, Matthew G. Vander Heiden, Sophie Vasseur

**Affiliations:** 1 Centre de Recherche en Cancérologie de Marseille (CRCM), Unité 1068, Institut National de la Santé et de la Recherche Médicale, Marseille F-13009, France; 2 Institut Paoli-Calmettes (IPC), Marseille F-13009, France; 3 Unité Mixte de Recherche (UMR 7258), Centre National de la Recherche Scientifique (CNRS), Marseille F-13009, France; 4 Université Aix-Marseille, Marseille F-13284, France; 5 Wolfson Wohl Cancer Research Centre, Institute of Cancer Sciences, College of Medical, Veterinary and Life Sciences, University of Glasgow, Garscube Estate, Switchback Road, Bearsden, Glasgow G61 1QH, UK; 6 Koch Institute for Integrative Cancer Research and Department of Biology, Massachusetts Institute of Technology, Cambridge, Massachusetts 02139, USA; 7 Aix Marseille Univ, INSERM, CRO2, Marseille F-13005, France; 8 Dana-Farber Cancer Institute, Boston, Massachusetts 02115, USA

## Abstract

Tissue architecture contributes to pancreatic ductal adenocarcinoma (PDAC) phenotypes. Cancer cells within PDAC form gland-like structures embedded in a collagen-rich meshwork where nutrients and oxygen are scarce. Altered metabolism is needed for tumour cells to survive in this environment, but the metabolic modifications that allow PDAC cells to endure these conditions are incompletely understood. Here we demonstrate that collagen serves as a proline reservoir for PDAC cells to use as a nutrient source when other fuels are limited. We show PDAC cells are able to take up collagen fragments, which can promote PDAC cell survival under nutrient limited conditions, and that collagen-derived proline contributes to PDAC cell metabolism. Finally, we show that proline oxidase (PRODH1) is required for PDAC cell proliferation *in vitro* and *in vivo*. Collectively, our results indicate that PDAC extracellular matrix represents a nutrient reservoir for tumour cells highlighting the metabolic flexibility of this cancer.

## Abstract (web summary)

Cancer cells adapt their metabolism to survive limited nutrient availability. Here, the authors show that in conditions of limited glucose or glutamine availability, pancreatic ductal adenocarcinoma cells can use collagen-derived proline to foster the TCA cycle and allow cell survival both *in vitro* and *in vivo*.

Pancreatic ductal adenocarcinoma (PDAC) has among the highest mortality rates across all forms of cancer^1^,^2^ and most patients survive <2 years post diagnosis^3^. The most common driver mutations in PDAC are activating mutations in *K-RAS*, observed in 95% of cases^4^, followed by loss of several tumour-suppressor genes (*INK4A* and *ARF*, *TP53*, *SMAD4*)^5^,^6^. PDAC tumours have a characteristic tissue architecture with cancer cells organized to form glandular structures surrounded by a tight desmoplasia composed of activated fibroblasts, immune cells, neurons and a dense collagen meshwork^7^,^8^. This fibrosis impedes vascularization, limits chemotherapy delivery^9^,^10^ and results in regions of reduced oxygen and nutrient delivery to the tumour cells^11^.

Mutant *K-RAS* promotes glucose metabolism to provide PDAC cells with ATP, reducing power (NADPH) and nucleoside precursors^12^. In addition, oncogenic K-RAS promotes enhanced glutamine catabolism to maintain redox status^13^, as well as extracellular protein uptake through macropinocytosis^14^. Yet because these tumours have poor nutrient delivery, the source(s) of nutrients for cancer cells in pancreatic tumours *in vivo* remains an open question. Given that pancreatic tumours contain a collagen-rich network that surrounds the cancer cells^15^,^16^, we hypothesized that collagen itself might represent an important nutrient source for tumour cells.

Collagen proteins are primarily synthesized by fibroblasts^17^ and remodelling normally occurs through breakdown into fragments by either extracellular or membrane-bound proteases present in the PDAC micro-environment including cathepsins and matrix metalloproteases^18^,^19^,^20^. Collagen fragments can induce downstream signalling in cells or be endocytosed^21^,^22^. Within fibroblasts, endocytosed collagen fragments are degraded by intracellular proteases into single amino acids, including proline, which is 25% of the total amino acid composition of collagen. In colorectal cancer, proline catabolism mediated by proline oxidase (POX, also called PRODH1) promotes tumour cell survival through either ATP production or autophagy induction, depending on whether the cancer cells are confronted with nutrient or hypoxic stress, respectively^23^. However, direct evidence of collagen uptake and degradation by pancreatic cancer cells is lacking, and whether proline from collagen breakdown is used to promote PDAC tumour cell survival or proliferation under nutrient stress is not known.

To test this possibility we used both human PDAC samples and the *Pdx1-Cre*;*KrasG12D;Ink4a−/−* (PKI) PDAC mouse model^24^, and demonstrate that PDAC cells catabolize collagen from their environment when nutrients *in vitro* are limited. We also show that collagen-derived proline is metabolized to fuel the tricarboxylic acid (TCA) cycle and contributes to PDAC cell survival under restrictive nutrient conditions, and that PRODH1 expression is important for PDAC tumour growth.

## Results

### PDAC tumours have an extracellular matrix rich in collagens

**Figure 1:**
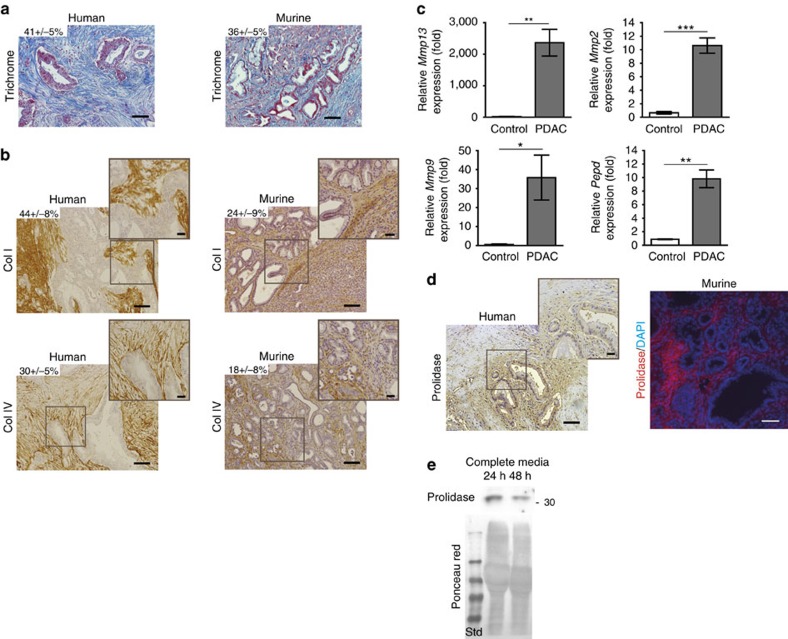
Collagen types I and IV and their associated collagenases are abundant in the stromal compartment of PDAC. (**a**) Collagen by Masson’s trichrome staining and (**b**) immunohistochemistry (IHC) of collagen proteins type I and IV in human and mouse tumour sections. Representative images and their relative insets from *n*=4 patients and *n*=3 Pdx1-Cre;*KrasG12D;Ink4a−/−* (PKI) mice are illustrated. The percentage of total collagen and collagen types I and IV staining intensity calculated relative to total tumour area, expressed as mean±s.e.m., is indicated. Scale bar, 100 μm. (**c**) mRNA levels of the collagen specific MMP 13, 9 and 2 and prolidase (*Pepd* gene) measured by quantitative RT–PCR in PDAC from PKI mice (*n*=3) versus control pancreases from *KrasG12D;Ink4a−/−* (KI) mice (*n*=3). Data are mean±s.e.m. *P<0.05, **P<0.01, ***P<0.001; two-tailed unpaired Student’s *t*-test. (**d**) Prolidase localization in human and PKI mice tumours revealed by IHC (left) or immunofluorescence (IF, right), respectively. Representative images from *n*=4 patients and from *n*= 3 PKI mice. Scale bar, 100 μm. (**e**) Prolidase levels in supernatant from PK4A cells cultured during 24 or 48 h with coated collagen I in complete media. Total loaded protein was determined by Ponceau red staining (Std: standard). Marker sizes are indicated in kDa. Photos are representative of *n*=3 independent experiments. Uncropped images of blots are shown in Supplementary Fig. 10. MMP, matrix metalloproteases; RT–PCR, PCR with reverse transcription.

We first confirmed the high collagen content of human and mouse PDAC tumours. Masson’s trichrome stained approximately 41% of the tumour area in human sections and 36% of PKI tumours (Fig. 1a). We then performed immunohistochemical (IHC) staining for collagens I and IV, the main collagen proteins found in human PDAC^15^,^25^,^26^. Antibodies against collagens I and IV each stained a large proportion of the tumour sections from both humans and mice (Fig. 1b). We also examined expression of collagen I- and IV-specific metalloproteases, namely matrix metalloprotease 13, 2 and 9, along with prolidase (encoded by *Pepd*), a protease specific for X-proline dipeptides (with X representing glycine or lysine) and found increased expression of these proteases in PDAC compared to control pancreas (Fig. 1c). While prolidase is described as an intracellular enzyme^27^, IHC staining using an antibody to prolidase overlapped with extracellular matrix (ECM) in both human PDAC and PKI tumours (Fig. 1d) and we also found that prolidase was excreted into the media from PDAC cells grown in culture (Fig. 1e). Together, these data confirm that PDAC tumours are enriched in collagen and suggest that enzymes are present in the tumour micro-environment that can generate collagen fragments and free amino acids that might be used as a fuel source by cancer cells.

### PDAC cells express proline metabolic enzymes

**Figure 2:**
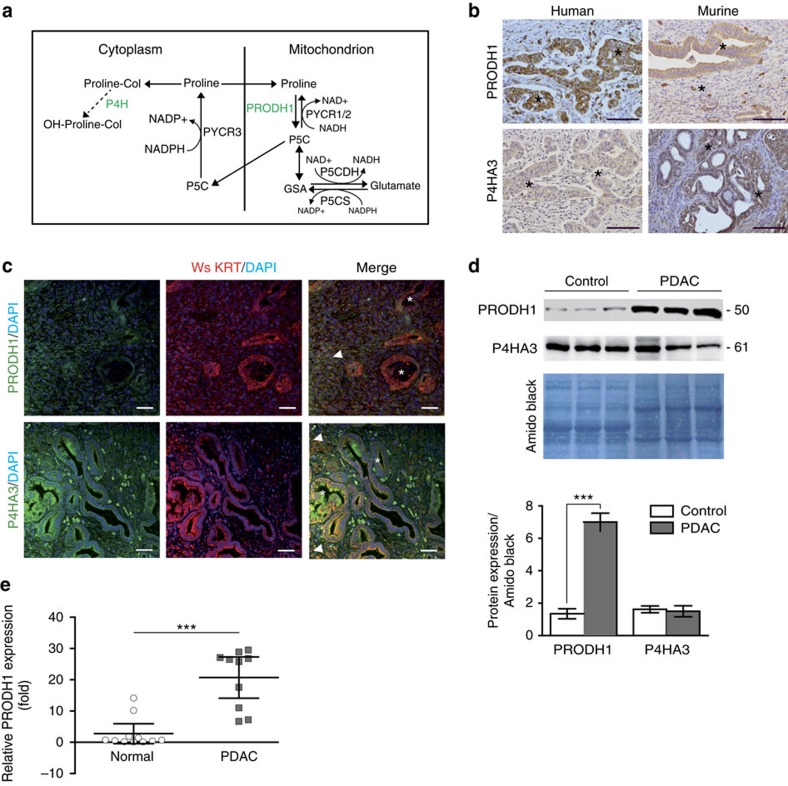
PDAC expresses proline metabolic enzymes. (**a**) Free proline is metabolized in the mitochondrial matrix, first by proline oxidase (PRODH1), to generate 1-pyrroline-5-carboxylic acid (P5C) before conversion into glutamic-γ-semialdehyde (GSA). P5C dehydrogenase (P5CDH) metabolizes GSA into glutamate and NADH. Free proline is synthesized from P5C via pyrroline carboxylases 1 and 2 (PYCR1, 2) in the mitochondrion and PYCR3 in the cytosol. Proline incorporated into collagen (proline-Col) can be modified into hydroxyproline (OH-proline) by prolyl-4-Hydroxylases (P4H). (**b**,**c**) Localization of PRODH1 and P4HA3 in human and PKI mice PDAC by IHC (**b**) and in epithelial compartment by IF co-staining with the wide-spectrum cytokeratin (Ws KRT) epithelial marker in PKI mice (**c**). Representative images from *n*=4 patients (**b**) and *n*=3 PKI mice (**b**,**c**). Tumour glands and disseminated cells are indicated by asterisks and arrows, respectively. Scale bar, 100 μm. (**d**) Quantification of PRODH1 and P4HA3 protein levels in PKI mouse PDAC and in KI mouse control pancreas revealed by western blot analysis (upper panel, with marker size indicated in kDa). Protein levels in each tissue samples (histograms, lower panel, from *n*=3 control KI mice and *n*=3 PDAC PKI mice) are normalized to total loaded-proteins (Amido black staining). Data expressed as mean±s.e.m. (***P<0.001; two-tailed unpaired Student’s *t*-test). Uncropped images of blots are shown in Supplementary Fig. 10. (**e**) Quantification of PRODH1 protein levels in normal human pancreas (*n*=11) and human PDAC samples (*n*=10). PRODH1 levels are normalized to one normal pancreas used as a reference. Data are illustrated as mean±95% CI, *** P<0.001; Mann–Whitney test. Images of human PDAC and normal pancreas macroarray are displayed in Supplementary Fig. 1. CI, confidence interval.

To begin to test the hypothesis that PDAC cells metabolize proline-derived from collagen breakdown, we measured expression of enzymes involved in metabolism of both free proline and proline in collagen (Fig. 2a)^28^. As assessed by IHC, PRODH1 and P4HA3 (the enzymatic subunit of P4H involved in proline hydroxylation in collagen synthesis) were found in tumour gland-like structures and we confirmed epithelial localization by co-staining with wide-spectrum cytokeratin (wsKRT) in murine tumour sections (Fig. 2b,c). Interestingly, we also noted cells disseminated in the stroma that are PRODH1 or P4HA3 positive (Fig. 2c). We then assessed overall tumour expression of PRODH1 and P4HA3 via immunoblot and found that PRODH1 is over-expressed (sevenfold) in PDAC relative to control tissue while P4HA3 protein levels are unchanged (Fig. 2d). Finally, we confirmed increased PRODH1 expression in human PDAC relative to normal pancreas via IHC (Fig. 2e) as well as expression in epithelial cells in all human pancreatic tumours examined (Supplementary Fig. 1). These data indicate enzymes involved in proline metabolism are expressed in PDAC cells.

### Nutrient-deprived PDAC cells take up collagens

**Figure 3:**
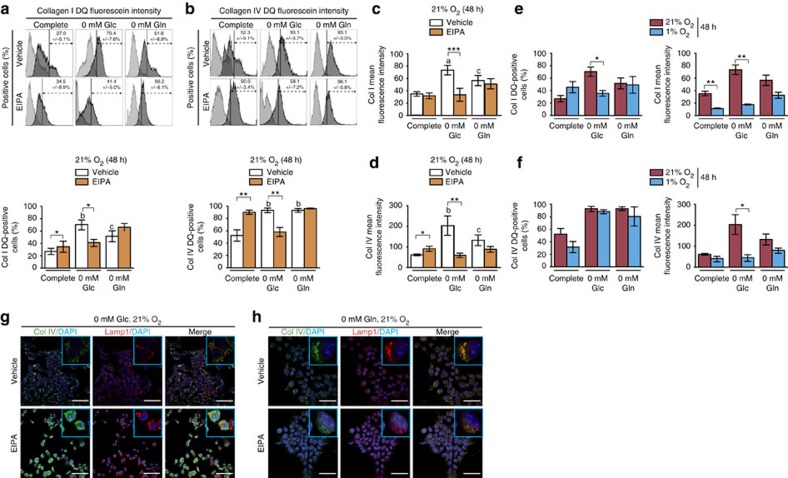
Nutrient stress induces PDAC cells to take up collagen through macropinocytosis-dependent and independent mechanisms. FACS analysis of collagen I or collagen type IV uptake by PK4A cells (**a** and **b** (upper) respectively). Cells were cultured 48 h in normoxia (21% oxygen (O_2_)) in complete (25 mM glucose, 4 mM glutamine), glucose-free or glutamine-free media with or without DQ-fluorescein-conjugated collagen I or IV (Col I or IV DQ). For each culture condition, either vehicle (DMSO) or 30 μM EIPA was added. Light grey peaks represent PKA cells not exposed to collagen I or IV DQ and dark grey peaks shifted to the right side reveal cells taking up collagen I or IV DQ. The percentage of positive cells in the dark grey shifted distributions, delimited by vertical bar, was indicated as mean±s.e.m. and represented as bar graph in charts below (lower **a** and **b** panels respectively). (**c**) Col I or (**d**) Col IV DQ MFI relative to each culture conditions during 48 h in normoxia. Data represent mean±s.e.m. (*n*=6 independent experiments). *P* values, indicated by asterisks, show statistical significance relative to respective culture media without EIPA, while letters indicate statistical significance relative to complete media without EIPA. (^c^*P*<0.05, ^b^*P*<0.01, ^a^*P*<0.001; two-tailed unpaired Student's *t*-test). (**e**,**f**) Comparison of the percentage (left panels) and MFI (right panels) between normoxic and hypoxic (1% oxygen (O_2_)) PK4A cells taking up Col I (**e**) or Col IV (**f**) DQ in each 48 h culture conditions. Data represent mean±s.e.m. (normoxia *n*=6 independent experiments; hypoxia *n*=3 independent experiments). *P<0.05, **P<0.01, two-tailed unpaired Student’s *t*-test. (**g**,**h**) Representative co-staining of Col IV DQ and Lamp1 lysosomal marker in PK4A cells cultured in glucose-free (**g**) or glutamine-free (**h**) media under normoxic conditions with or without EIPA. Insets show magnification of Col IV, Lamp1 or Col IV/Lamp1 (merge) PKA4 cells in each condition. Images are representative of *n*=3 independent experiments. Scale bar: 50 μm. FACS, fluorescence-activated cell sorting. MFI, mean fluorescence intensity.

Since the dense stromal reaction impedes nutrient delivery to tumour cells, we reasoned that pancreatic cancer cells might express matrix remodelling and proline catabolic enzymes to allow for the utilization of ECM proteins as a source of amino acids under nutrient-deprived conditions. K-RAS mutant tumour cells starved of essential amino acids are known to activate macropinocytosis^29^, a phenomenon that involves the nonspecific uptake of extracellular material into vesicles that fuse with lysosomes, allowing subsequent metabolism of the internalized products^30^. To confirm that this process is active in PK4A cells, we utilized DQ-fluorescein-conjugated collagen I or IV (collagen I or IV DQ). Because the fluorophore in these molecules is quenched within the intact protein, fluorescence is only observed following protein breakdown and allows for the assessment of both protein uptake and cleavage^31^. Collagens I and IV uptake were readily observed under both normoxic and hypoxic culture conditions (Fig. 3a–f and Supplementary Fig. 2a–d). In particular, uptake of collagens I and IV were both dramatically increased in normoxia upon deprivation of either glucose or glutamine; however, this increase was not observed to the same extent in hypoxic cells (Fig. 3a–d and Supplementary Fig. 2a–d). Interestingly, the macropinocytosis inhibitor 5-*N*-ethyl-isopropyl-amiloride (EIPA)^14^ blunted the increase in collagens I and IV DQ fluorescence observed following glucose limitation, but resulted in increased uptake in complete media and had no effect under glutamine-limited conditions (Fig. 3a–d). Culturing PDAC cells under hypoxic conditions did not enhance collagen uptake compared to culture in normoxia with or without nutrient deprivation (Fig. 3e,f). Moreover, hypoxia blunts the effect of EIPA when glucose is low (Supplementary Fig. 2a,d). Taken together, these data demonstrate that glucose or glutamine deprivation promotes uptake and breakdown of collagens I and IV protein by PDAC cells, with macropinocytosis playing a key role under basal and glucose-limited conditions while other mechanisms are likely involved in collagen breakdown in PDAC tumours under glutamine-limited conditions.

After collagen is taken up from the surrounding milieu, further metabolism is necessary to breakdown the proteins and utilize the component amino acids. To ascertain the subcellular location of collagen in nutrient-deprived PK4A cells, we performed co-immunofluorescence (IF) with the lysosomal membrane protein marker LAMP1 and we demonstrated collagen localization to punctate LAMP1-positive structures in glucose-starved cells, consistent with some collagen IV DQ degradation within lysosomes (Fig. 3g). EIPA-induced collagen spreading throughout the cytoplasm was also observed, implying extra-lysosomal degradation of collagen may also be active (Fig. 3g, EIPA). Consistent with our above findings, collagen localization within lysosomal vesicles was not affected by EIPA under glutamine starvation (Fig. 3h) and no differences were observed between normoxic and hypoxic conditions (Supplementary Fig. 2e,f). Thus, under nutrient limited conditions PK4A cells increase collagen uptake, but through both macropinocytosis-dependent and independent mechanisms. Macropinocytosis appears to be a route of collagen uptake following glucose starvation under normoxia, while macropinocytosis-independent pathways appear to allow collagen uptake by these cells following glutamine starvation or in hypoxia.

One mechanism for collagen uptake other than macropinocytosis is endocytosis mediated by the collagen receptor uPARAP/Endo180. This process is found in tissues with enhanced matrix remodelling, including many tumours^32^. We found that uPARAP/Endo180 is highly expressed in PDAC compared to control pancreases in mice (Supplementary Fig. 3a,b) and localized to gland-like structures in both mouse and human PDAC samples (Supplementary Fig. 3c). Thus, in addition to macropinocytosis as a mechanism for collagen uptake, uPARAP/Endo180 could support collagen uptake as well. Interestingly, this receptor has been previously associated with migratory processes in pancreatic tumours suggesting it plays an active role in remodelling the ECM in pancreatic cancer^33^.

### Collagen and proline promote cell survival and proliferation

**Figure 4:**
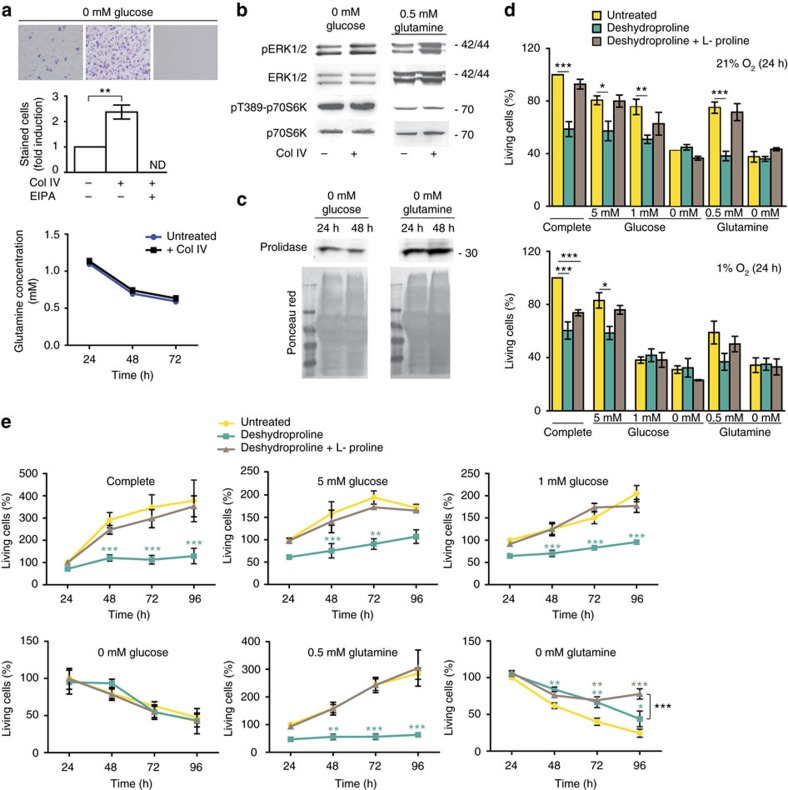
Collagen uptake promotes survival and proline promotes both survival and proliferation. (**a**) PK4A cell survival in indicated media supplemented with vehicle or water-soluble collagen IV alone or in combination with EIPA treatment (upper). After treatments, cells were submitted to crystal violet assay. (upper) Representative crystal violet images (*n*=3 independent experiments). (middle) Crystal violet intensity, expressed as mean±s.e.m. (*n*=3 independent experiments) is presented as fold-induction relative to intensity in glucose-free condition without EIPA and without col IV. ND: Value below detection level. **P<0.01, two-tailed unpaired Student’s *t*-test. (lower) Glutamine concentration in media of PK4A cells cultured in glucose-free medium supplemented or not with water-soluble collagen IV over 72 h. Data represent mean±s.e.m. (*n*=3 independent experiments). Two-way ANOVA followed by Bonferroni *post-hoc* test revealed no significant difference in PK4A glutamine uptake between the two culture conditions. (**b**) ERK1/2 and p70S6K phosphorylation status in PK4A cells cultured with or without water-soluble collagen IV in indicated media during 72 h. (**c**) Prolidase levels in supernatant from PK4A cells cultured with coated collagen I in indicated media. Total protein loading was determined by Ponceau red staining. (**b**,**c**) marker sizes are indicated in kDa. Photos are representative of *n*=2 (**b**) and *n*=3 (**c**) independent experiments. Uncropped images of blots are shown in Supplementary Fig. 10. (**d**) Survival of PK4A cells cultured under normoxic (21% O_2_) or hypoxic (1% O_2_) conditions (upper and lower charts, respectively) in indicated nutrient conditions, in media alone or supplemented with either DHP (1 mM) or with DHP and L-proline (500 μM). Living cells, determined 24 h after treatment, are expressed as percentage of untreated cells in complete media (mean±s.e.m., *n*=4 independent experiments). *P<0.05, **P<0.01, ***P<0.001; two-tailed unpaired Student's *t*-test. (**e**) Proliferation of PK4A cells cultured for 24–96 h under normoxia as in **d**. Data are mean±s.e.m., *n*=4 independent experiments. *P* value, is relative to corresponding untreated value at each time point.*P<0.05, **P<0.01, ***P<0.001; two-way ANOVA followed by Bonferroni *post-hoc* test. ANOVA, analysis of variance.

On the basis of our previous observations surrounding the active uptake and breakdown of collagen proteins, we hypothesized that this could support tumour cell survival in response to nutrient deprivation, a condition thought to be present in the pancreatic tumour micro-environment. To test this possibility, we cultured PK4A cells for 72 h without glucose with or without collagen supplementation, before re-introduction of complete media for 24 h and assessment of cell survival and found that both soluble or coated collagen significantly increased pancreatic cell survival (Fig. 4a upper panel: two-tailed unpaired Student’s *t*-test, *P*=0.0075, Supplementary Fig. 4a,b). EIPA reduced cell survival, further suggesting that macropinocytosis of collagen (along with other nutrients) is involved in cell survival rescue under glucose-limited conditions (Fig. 4a). Interestingly, over the 3 days of glucose starvation, glutamine concentration decreases to low levels creating a concomitant glucose and glutamine deprivation at 72 h of culture (Fig. 4a lower panel).

Looking downstream of oncogenic K-RAS, we found that under glucose and glutamine-depleted conditions, ERK1/2 phosphorylation is increased in the presence of collagen (Fig. 4b, upper gels). In K-RAS mutant cells, essential amino-acid deprivation strongly represses the mTORC1 pathway, but the uptake of extracellular proteins can reactivate signalling and result in phosphorylation of downstream targets such as S6K1 (ref. 29). However, collagen addition in glucose-free or glutamine-reduced conditions does not appear to impact downstream mTORC1-signalling compared to basal levels observed without collagen (Fig. 4b, lower gels). Thus, collagen both rescues pancreatic tumour cell survival and increases ERK1/2-pathway activation following glucose and/or glutamine shortage without engaging the mTORC1-signalling cascade.

As the proline catabolic enzyme PRODH1 is over-expressed in PDAC compared to control pancreas tissue (Fig. 2) and collagen breakdown can be a source of proline (Fig. 3), we investigated if PK4A cells could use collagen-derived proline to maintain their survival and/or proliferation under nutrient limitation. We first confirmed that the extracellular secretion of prolidase we previously observed in murine and human tumours (Fig. 1d,e) was also present under low glucose and glutamine conditions (Fig. 4c). This suggests external degradation of collagen might be used as a source of free proline in case of nutrient deprivation.

To assess the possible fates of this free proline and gain initial insight into how it might promote survival and/or proliferation in some conditions, we utilized [U-^14^C]-proline to trace the fate of proline into all major biomass and metabolite compartments of PDAC cells^34^. Interestingly, we found that proline carbon primarily contributes to biomass through incorporation into protein, although a small contribution of proline carbon to other macromolecules was also observed (Supplementary Fig. 5).

Although relatively small, the presence of proline-derived carbon in non-protein metabolic compartments as well as the increased expression of PRODH1 in PDAC (Fig. 2d) led us to more closely examine proline metabolism under nutrient limited conditions. We cultured PK4A cells in either complete media, low glucose (0, 1, 5 mM) or low glutamine (0, 0.5 mM) conditions, with or without free proline, in the presence or absence of deshydroproline (DHP), a competitive inhibitor of PRODH1. Exposure to DHP diminishes both cell survival and proliferation under some nutrient limited conditions (5, 1 mM glucose and 0.5 mM glutamine), whereas addition of excess free proline rescued the survival and proliferation of the DHP-treated cells (Fig. 4d upper panel, Fig. 4e). In hypoxic conditions, inhibition of PRODH1 does not impact cell survival to the same extent as it does under normoxia (Fig. 4d lower panel). Taken together, these data argue that proline catabolism through PRODH1 can support cell survival and proliferation especially in glucose and glutamine-restricted conditions.

### Proline degradation under low glutamine limits glycolysis

**Figure 5:**
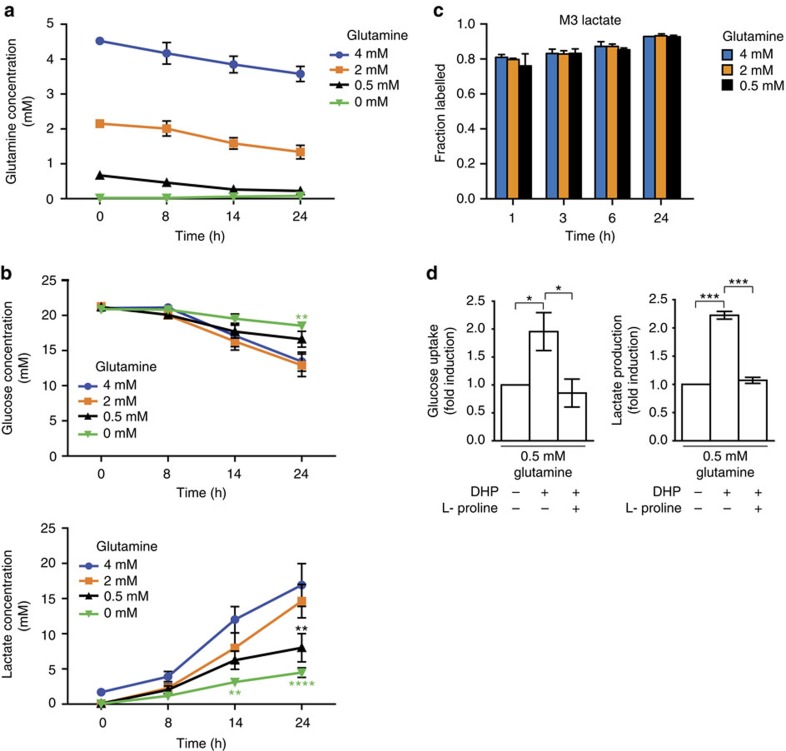
Proline catabolism diminishes glycolytic flux under glutamine deprivation. (**a**) Glutamine and (**b**) glucose (upper) and lactate (lower) concentration in supernatant of subconfluent PK4A cells cultured for 8–24 h under gradual decrease of glutamine concentration (4, 2, 0.5 and 0 mM). Data are mean±s.e.m., *n*=3 independent experiments. *P* value, indicated by green and dark asterisks in **b**, is relative to glucose or lactate value in 4 mM glutamine at each time point. **P<0.01, ***P<0.001; two-way ANOVA followed by Bonferroni *post-hoc* test. (**c**) Fractional labelling (M3) of lactate from U-^13^C-glucose at 1, 3, 6 and 24 h under various glutamine concentrations (4 mM, 2 mM, 0.5 mM). All data were presented as mean±s.e.m. (*n*=3 independent experiments). (**d**) Glucose consumption and lactate production by PK4A cells cultured during 24 h in 0.5 mM glutamine media alone, or supplemented with DHP (1 mM) or with DHP and L-proline (500 μM). Data are presented as fold induction (mean±s.e.m., *n*=3 independent experiments) relative to untreated media values. *P<0.05, ***P<0.001; two-tailed unpaired Student’s *t*-test. ANOVA, analysis of variance.

Glutamate serves as the common metabolic intermediate between glutamine and proline metabolism^35^. We confirmed PDAC cells *in vitro* actively deplete glutamine from the culture media (Fig. 5a). However, as has been demonstrated in other contexts^36^, cells cultured in low glutamine (0.5, 0 mM) consume less glucose than cells cultured in high glutamine medium (4 mM) and also show decreased lactate production (Fig. 5b). Because lactate is derived exclusively from glucose (Fig. 5c and Supplementary Fig. 6a,b), this decrease in lactate suggested that restricting glutamine availability can decrease glycolytic flux.

We next evaluated how proline catabolism influenced the metabolic status of glutamine-restricted PDAC cells. Interestingly, blocking proline catabolism with PRODH1 inhibition significantly increased glycolytic activity of PK4A cells cultured under low glutamine conditions, as shown by an increased glucose consumption and lactate production after DHP treatment (Fig. 5d: two-tailed unpaired Student’s *t*-test, *P*=0.0481 and *P*<0.0001, respectively). This increase in glucose consumption and lactate production in response to DHP was inhibited when free proline was added back to glutamine-low culture media, supporting the notion that proline degradation may serve as an alternative nutrient source to glucose when glutamine is limiting.

### Proline fuels tricarboxylic acid (TCA) cycle metabolism

**Figure 6:**
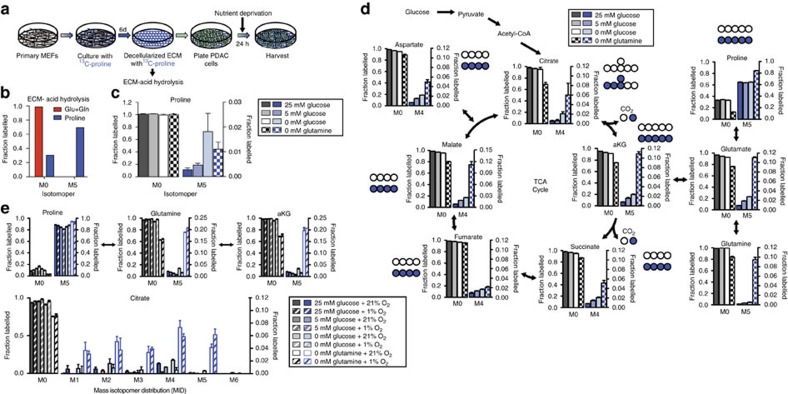
Proline supports TCA metabolism under nutrient limited conditions. (**a**) Schematic representation of the protocol used to create extracellular matrix (ECM) containing U-^13^C-proline. MEFs: mouse embryonic fibroblasts. (**b**) GCMS analysis of acid hydrolysis of de-cellularized ECM proteins from experiment outline in part a. Data is from *n*=1 experiment and is representative of *n*=2 experiments. (**c**) Fractional labelling of free U-^13^C-proline pools inside PK4A cells after 24 h on labelled ECM under indicated nutrient conditions. (**d**) U-^13^C-proline tracing by GCMS analysis into TCA intermediates in PK4A cells for 24 h under indicated nutrient conditions. M0 represents the unlabelled molecule with each increasing number representing another labelled carbon. Empty circles represent unlabelled ^12^C-species, while filled circles represent ^13^C-labelled carbons. For **c** and **d** data are mean±s.e.m. of *n*=3 biological replicates and representative of at least *n*=2 independent experiments. (**e**) Fractional labelling of proline from U-^13^C-proline after 24 h at the indicated conditions at normoxia (21% O_2_) and hypoxia (1% O_2_) (upper panels). All data are presented as mean±s.e.m. *n*=3 per condition. Complete mass isotopomer distribution (MID) of citrate from U-^13^C-proline after 24 h at the indicated conditions at normoxia (21% O_2_) and hypoxia (1% O_2_) (lower panel). All data are presented as mean±s.e.m. *n*=3 per condition. GCMS, gas chromatography-mass spectrometry.

**Figure 7:**
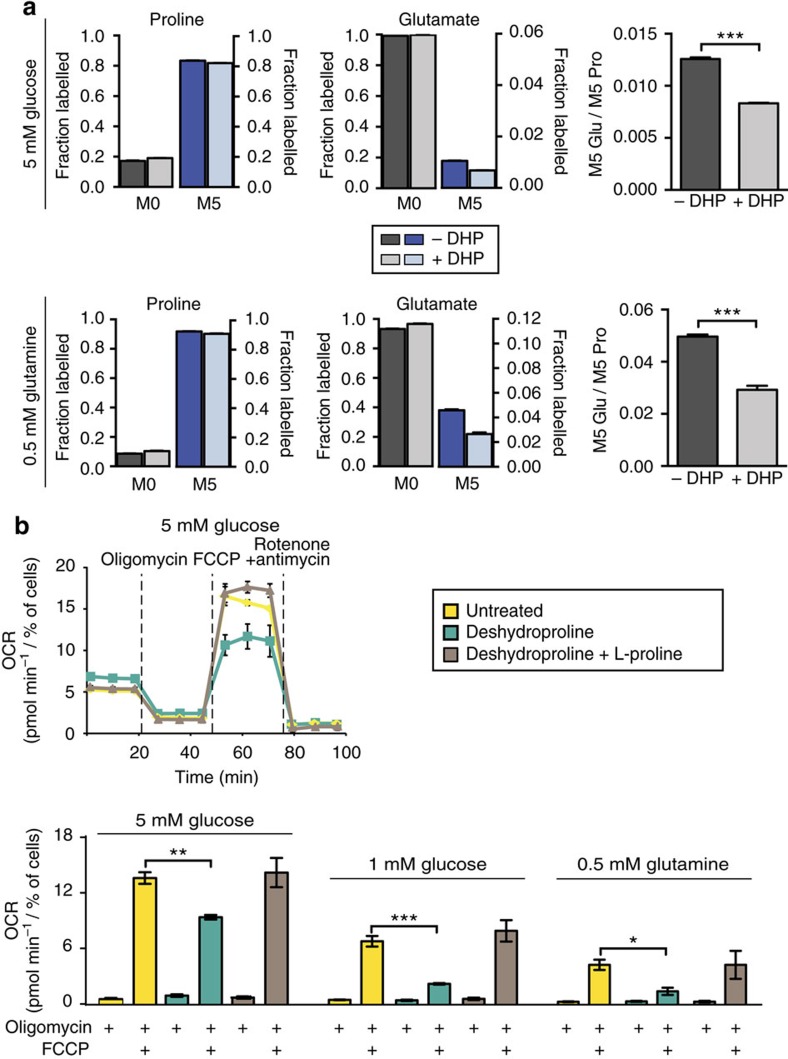
DHP limits production of proline-derived glutamate and mitochondrial respiration. (**a**) Fractional labelling of proline and glutamate from U-^13^C-proline after 24 h at 5 mM glucose (upper charts) or 0.5 mM glutamine (lower charts) media alone or supplemented with DHP (1 mM). Ratio of M5 glutamate (Glu) to M5 proline (Pro) at 5 mM glucose or 0.5 mM glutamine are presented next to labelled fractions. All data are presented as mean±s.e.m. *n*=3 per condition. ***P<0.001; two-tailed unpaired Student’s *t*-test. (**b**) Representative oxygen consumption rate (OCR) profile of PK4A cells cultured in 5 mM glucose media alone, or supplemented with DHP (1 mM) or with DHP and L-proline (500 μM) (upper panel). Sequential injections of 1 μM oligomycin, 1 μM FCCP and 0.5 μM rotenone/antimycin A mix are indicated. Maximal respiration was determined under indicated limited-nutrient conditions (lower panel). Data are mean±s.e.m., *n*=3 independent experiments. *P<0.05, ***P<0.001; two-tailed unpaired Student’s *t*-test.

To confirm that proline catabolism is active and to begin to address the fate of catabolized proline in PDAC cells we performed a series of tracing experiments utilizing U-^13^C-proline. We first generated U-^13^C-proline-containing ECM using primary mouse embryonic fibroblasts (MEFs) (Fig. 6a,b and Supplementary Fig. 7a,b) to assess whether ECM protein-derived proline could be harvested by PDAC cells under nutrient limited conditions. Assessment of the amino acid composition demonstrates that the proportion of individual amino acids in ECM differs from that found in Dulbecco’s modified Eagle’s medium (DMEM), and that ECM contains relatively larger quantities of proline and glycine (Supplementary Fig. 7c). After plating cells on this labelled matrix, we switched to media with limited glucose and glutamine concentrations for 24 h (Fig. 6a). We observed accumulation of free U-^13^C-proline from the labelled ECM into PDAC cells when nutrients were deprived (Fig. 6c), confirming that PK4A cells are able to extract proline from ECM. To address the fate of catabolized free proline under the same nutrient-deprived conditions, we traced U-^13^C-proline in these cells. Consistent with the ability of proline to rescue cell viability and proliferation under nutrient limitation, label from proline was found in all TCA cycle intermediates in PDAC cells, with labelling patterns indicating multiple turns of the TCA cycle, under conditions where glucose or glutamine were limited (Fig. 6d and Supplementary Fig. 7d). These contributions are slightly decreased under hypoxia but under glutamine-free conditions we observe increased M+5 labelling of citrate, raising the possibility that proline can substitute as a substrate for reductive α-ketoglutarate metabolism, as it was previously described for glutamine in hypoxic or anchorage independent culture conditions^37^,^38^ (Fig. 6e and Supplementary Fig. 8a). Furthermore, we observe inhibition of proline metabolism to glutamate by 1 mM DHP in the presence of 500 μM U-^13^C-proline under both 0.5 mM glutamine (with 25 mM glucose) and 5 mM glucose (with 4 mM glutamine) conditions (Fig. 7a and Supplementary Fig. 8b). Under these conditions DHP also decreases oxygen consumption in cells treated with oligomycin and carbonyl cyanide-4-(trifluoromethoxy) phenylhydrazone (FCCP) (the combination of which enables the full oxygen respiratory capacity of cells), while addition of excess proline restores full respiration capacity (Fig. 7b). Taken together, these data suggest collagen-derived proline promotes cell survival and can contribute to proliferation under nutrient limitation at least in part by supporting TCA cycle metabolism and cellular respiration.

### PRODH1-mediated proline metabolism promotes PDAC growth

**Figure 8:**
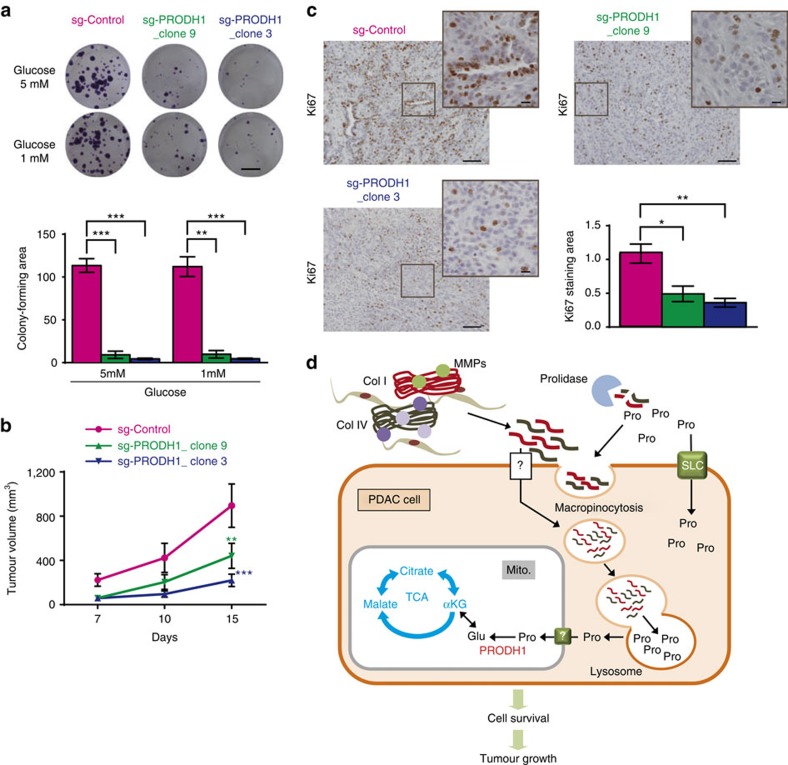
PRODH1 inhibition limits pancreatic cancer cell resistance to nutrient limitation and impedes pancreatic tumour growth. (**a**) Representative clonogenic assay and quantification of sg-Control and sg-PRODH1_clone9 and _clone3 PK4A cells colony-forming area in 5 and 1 mM glucose media. Data are mean±s.e.m. (*n*=3 independent experiments) and are expressed in percentage of 5 mM glucose sg-Control value. **P<0.01, ***P<0.001; two-tailed unpaired Student's *t*-test. Scale bar: 5 mm. (**b**) Tumour regression measured in sg-PRODH1_clone9 and _clone3 mice as compared to sg-Control one. Mice were subcutaneously implanted with sg-Control or sg-PRODH1_clone9 or _clone3 PK4A cells and caliper measurement of tumour size was performed over 15 post-implantation days. Data are mean±s.e.m. (*n*=10 mice per group). *P* value, indicated by green and blue asterisks, is relative to sg-Control tumour volume after 15 days post-implantation. **P<0.01, *** *P*< 0.001; two-way ANOVA followed by Bonferroni post-hoc test. (**c**) Ki67 IHC staining in sg-Control, sg-PRODH1_clone9 or _clone3 subcutaneous pancreatic tumour sections. Representative images from *n*=4 mice per group are illustrated. Scale bar: 100 μm. Chart represents quantification of Ki67 stained area/ quantified tumor area (*n*=7 fields quantified per tumour). *P<0.05, **P<0.01; two-tailed unpaired Student’s *t*-test. (**d**) Schematic summary. Upon nutrient limitation PDAC cells degrade collagen-rich PDAC extracellular matrix proteins and take up free proline through membrane transporters (SLC) as well as collagen fragments through macropinocytosis-dependent and independent processes. Internalized vesicles fuse with lysosome and collagen I and IV (ColI and ColIV) fragments undergo further degradation towards free amino acids. Collagen-derived proline (Pro) is further metabolized by the tricarboxylic acid (TCA) cycle to promote PDAC cell survival. MMPs: Matrix metalloproteases, Glu: Glutamate, α-KG: α-ketoglutarate, Asp: Aspartate. Mito: Mitochondria. ANOVA, analysis of variance.

We finally evaluated the requirement for PRODH1-mediated proline catabolism on pancreatic tumour cell phenotypes *in vitro* and *in vivo*. We generated two PK4A cell lines with CRISPR-Cas9-mediated disruption of the PRODH1 gene, in which the PRODH1 protein was decreased up to 80% (Sg-PRODH1_clone9 and Sg-PRODH1_clone3) as compared to sg-Control_PK4A cells (sg-Control) (Supplementary Fig. 9a). We cultured these cell lines at low confluence under complete media or glucose-restricted conditions and found that disruption of PRODH1 not only drastically limited the clonogenic capacity of PK4A cells in full, but also in low glucose conditions (Fig. 8a and Supplementary Fig. 9b). We then tested the ability of Sg-Control PK4A cells and Sg-PRODH1 PK4a cells to form tumours in nude mice. Strikingly, the tumorigenic potential of PK4A cells was dramatically impaired when PRODH1 was inhibited (Fig. 8b) and Ki-67 staining was lower in tumours with disrupted PRODH1 expression compared to control tumours (Fig. 8c). Taken together, these data indicate that PRODH1 expression promotes pancreatic tumour growth, and argues that acquisition of proline from the metabolism of collagen in the tumour environment promotes PDAC tumour cell survival.

## Discussion

The metabolic flexibility of tumour cells is widely recognized^39^; however, the tissue context can greatly constrain which nutrients and pathways are utilized by cancer cells^40^,^41^,^42^,^43^. PDAC tumours cells are embedded within a tight collagen-rich desmoplasia, lowering vascularization, nutrients and oxygen supply to tumour cells and severely restricting the pool of available substrates to support survival or even growth. However, the collagen matrix provides a potential source of some amino acids, including proline and we find that tumour cells take up and degrade ECM proteins including collagens I and IV to support TCA cycle metabolism under nutrient limited conditions (Fig. 8d). This use of ECM proteins illustrates how cancer cell can exploit their immediate environment and utilize available substrates when other fuels are limiting.

Recent work has highlighted the importance of the bulk uptake of soluble protein via mutant *K-RAS*-driven macropinocytosis as a source of amino acids to support survival and proliferation under times of nutrient stress *in vitro*^14^,^29^,^44^. Consistent with these results, we found macropinocytosis was major contributor to the uptake of collagens I and IV proteins, presumably along with other nutrients, at least under conditions of glucose deprivation. Interestingly, however, this process did not seem to play a role under hypoxia or glutamine-restricted condition. This raises the possibility that parallel mechanisms of collagen uptake involved in cancer progression^45^, including the similarly upregulated uPARAP/Endo180 pathway, actively participate in metabolism by internalizing intact or partially degraded collagen fragments^22^,^46^. In addition, we demonstrate that collagen uptake promotes PK4A cell survival under nutrient deprivation and leads to the activation of the ERK1/2-pathway (Fig. 4). A signalling connection between collagen and ERK activation was previously identified, although mainly associated with cancer cell migration and invasion^47^,^48^. Thus our data now provide an additional layer of the interplay between collagen, ERK signalling and cell survival. The absence of mTORC1 activation by collagen degradation underscores the mechanisms exploited by PDAC cells to favour the active uptake and use of ECM proteins. More specifically, mTORC1 signalling has been shown *in vitro* to block utilization of exogenous protein-derived amino acids and inhibition of this complex promotes continued division under nutrient limited conditions *in vivo*^29^.

Our work shows that PDAC cells use proline under nutrient deprivation to sustain viability via TCA cycle metabolism (Fig. 6). Previous studies showed that glutamine feeds the TCA cycle to produce proline and that PRODH1 activity promotes colon tumour cell survival under nutrient stress via autophagy and/or ATP production^35^,^49^. In PDAC, we also find that proline degradation can fuel glutamine synthesis primarily in glutamine-free conditions. More broadly, however, increased proline degradation and entry into the TCA cycle for oxidation occurs in the nutrient limited condition examined here. Thus, proline appears to be able to substitute at least temporarily for glucose and glutamine to promote PDAC cell survival under extremes of nutrient limitation, likely by generating ATP. Hence, we propose that, as the main reservoir of proline in PDAC, collagen represents an important nutrient pool for pancreatic tumours, although it is important to recognize that insoluble ECM proteins do not necessarily reflect a well-balanced diet of essential amino acids (Supplementary Fig. 7c) to promote long-term proliferation in all PDAC cells. Nevertheless, the metabolism of ECM-derived proline to replenish TCA intermediates may be a critical mechanism to promote the survival of the frequently chemo-resistant and slow-dividing cells nearest to tumour cores. Furthermore, some degree of proline metabolism through PRODH1 appears necessary for proliferation under even nutrient replete conditions and enables PDAC tumour growth *in vivo* in addition to the aforementioned survival benefit. This raises the possibility then that targeting proline metabolism specifically through the inhibition of catabolic enzymes such as PRODH1, might both slow overall tumour growth and compromise the survival of more resilient cancer cells within the tumour.

## Methods

### Human samples

Formalin-fixed, paraffin-embedded tissue samples of PDAC were obtained from 18 patients who had undergone surgery at the Department of Digestive Surgery, North Hospital, Public Assistance of Marseille’s Hospitals, France. Before surgery, all patients had signed an informed consent form that had been approved by the local ethics committee (Agreement reference of CRO2 tissue collection: DC-2013-1857). Histological examination confirmed a diagnosis of PDAC in all cases. Tumour staging was performed according to the International Union Against Cancer TNM System (6th edition).

### Mouse strains and tissue collection

Male *Pdx1-Cre;LSL-Kras*^*G12D*^*;Ink4a/Arf*^*fl/fl*^ (PKI) PDAC-bearing mice, and male *LSL-Kras*^*G12D*^*;Ink4a/Arf*^*fl/fl*^(KI) control littermates were obtained as previously described^50^. After mice were killed at the age of 9 weeks, pieces of tumour or control pancreas were either fixed in 4% (wt/vol) formaldehyde, snap-frozen in cold isopentane for further analysis, or directly homogenized in 4 M guanidinium isothiocyanate lysis buffer for efficient pancreatic RNA extraction, according to previously published protocols^51^. All animal care and experimental procedures were performed in agreement with the Animal Ethics Committee of Marseille under reference 01527.02.

### Cell culture conditions

PK4A cell line was isolated from PKI murine PDAC. Briefly, PDAC from PKI mice were chopped into small pieces with a razor blade. Cell homogenate was successively incubated in Hanks' balanced salt solution (HBSS) solutions containing 0.9 g of glucose with collagenase (30 min at 37 °C) and then trypsin (5 min at 37 °C). Then, cells were plated and maintained in Glutamax 25 mM glucose DMEM supplemented with 10% (vol/vol) fetal bovine serum (FBS) (Gold, GE Healthcare), 1% antibiotic/antimycotic (anti/anti) solution for several passages before examination for epithelial markers^50^. PK4A cells were used from passages 18 to 28 and were demonstrated to be negative for mycoplasma contamination. For experiments, glucose and glutamine concentrations in the base DMEM media (using commercial L-glutamine, DMEM without glutamine, 25 mM glucose or DMEM without glucose, 4 mM glutamine) were modified to the indicated concentrations and the media was supplemented with 10% dialysed FBS (SH30079, Hyclone), and complete media (25 mM glucose, 4 mM glutamine DMEM) was supplemented with 10% filtered FBS (SH30070, Hyclone), all media supplemented with 1% anti/anti. Cells were cultured at 37 °C at 21% oxygen in a 5% CO2 incubator. For hypoxic experiments, cells were placed in a Whitley Hypoxic WorkStation (Don Whitley Scientific) at 1% oxygen concentration, 5% CO_2_, 37 °C. Unless otherwise stated, all cell culture reagents were purchased from Life Technologies.

### Collagen solubilization

Collagen type IV from Engelbreth–Holm–Swarm murine sarcoma basement membrane (Sigma-Aldrich, #C0543) was solubilized in sterile water through heating/cooling cycles. Briefly, lyophilized collagen was resuspended in water and heated 2 h at 60 °C. Dissolved material was then cooled down at 4 °C O/N, re-heated 1hr at 60 °C (which separates, but does not fragment collagen fibrils) before complete resuspension and subsequent use.

### Reverse transcription and quantitative real-time PCR

RNA quality was assessed with the RNA Nano Chip kit (Agilent) on an Agilent Bioanalyser and treatment with DNase was performed using the RNase-free DNase set (Qiagen). Five microgram of total RNA from each sample was used to synthesize cDNA with the PrimeScript RT reagent kit (Promega) and provided oligo-dT primers, according to the manufacturer’s instructions. Quantitative PCR reactions were performed with specific primers listed in Supplementary Table 1 and the GoTaq qPCR master mix kit (Promega) using the Mx3005P Stratagene system. Differential expressions of transcripts of interest were calculated in relation to the 36B4 housekeeping transcript.

### Protein extracts and western blots

Samples from PDAC and control pancreases from PKI and KI mice were lysed (10% N-Deoxycholate, 0.1% SDS, 1% Triton X-100, 10 mM Tris pH 8, 140 mM NaCl, phosphatases/proteases inhibitors (Cocktail from Sigma-Aldrich), 1% phenylmethylsulfonyl fluoride, 1 mM Sodium fluoride (NaF), 100 μM Sodium orthovadanate (Na_3_VO_4_), 40 mM beta glycerophosphate), before centrifugation (14,000 r.p.m., 10 min, 4 °C). Supernatants were collected and protein concentration evaluated using BioRad Protein assay. For soluble collagen-induced signalling studies, 5 × 10^5^ PK4A cells were seeded on 6 cm plates and cultured 72 h in reconstituted, no glucose or 0.5 mM glutamine DMEM with or without water-dissolved collagen IV (20 μg ml^−1^) before protein extract preparation. To evaluate PRODH1 levels in sg-PRODH1_ PK4A clones, 5 × 10^5^ cells were seeded on 6 cm plates 24 h before protein extract preparation. Whole cell protein extracts (75-100 μg per lane) were resolved by SDS–polyacrylamide gel electrophoresis (SDS–PAGE) using a 10% (vol/vol) acrylamide gel and transferred onto 0.2 μm nitrocellulose membranes (GE Healthcare). After a blocking step (5% (wt/vol) non-fat milk in TBS), primary antibodies were incubated O/N at 4 °C (in non-fat milk, 5% in TBS 0.1% Tween). Following primary antibodies were used: PRODH1 (1:500, Sigma-Aldrich, #SAB2501795), P4HA3 (1:500, Abcam, #ab101657), pERK1/2 (1:500, Sigma-Aldrich, #M8159), ERK1/2 (1:2,000, Sigma-Aldrich, #M5670), prolidase (1:750, Novus biological, #H00005184-M01), pT389-p70S6Kinase (1:400, Cell Signaling, ##9234), p70S6Kinase (1:200, Santa Cruz Biotechnologies, #sc-8418), uPARAP/Endo180 (1:700, Abcam, #ab70132) and β-actin (1:10,000, Sigma-Aldrich, A5316). For western blot of decellularized ECM, ECM extracts were resolved by SDS–PAGE using a 6% (vol/vol) acrylamide gel. An amount of 30 μl of decellularized ECM taken up in 3 × SDS loading dye (200 ul per well on six-well dish) was loaded and compared with 20 μg of lysate from control MEFs. After transfer to PVDF membrane and blocking in 5% BSA in TBS+0.1% Tween, blotting was performed using primary antibodies against Collagen I (1:1,000, Millipore, #AB765P), Fibronectin (1:10,000, a gift from Richard O. Hynes, Koch Institute for Integrative Cancer Research and Howard Hughes Medical Institute, Massachusetts Institute of Technology, Cambridge, Massachusetts, USA. Antibody was used as described before^52^) and Hsp90 (1:1,000, Cell Signaling, #4877). For excreted prolidase analysis, 6-well plates were coated with 20 μg/cm2 collagen I. Briefly, collagen I (0.1% in 1 N acetic acid, Sigma #C8919) was diluted 10 times in PBS, and distributed in six-well plates. Collagen was fibrillated 2 h at 37 °C. After complete evaporation, wells were washed in PBS and dried O/N before use. 5 × 10^5^ PK4A cells were seeded on collagen coated plates for 24 to 48 h in complete, no glucose or no glutamine medium. Medium was collected at each time point, centrifuged (2,000 r.p.m., 3 min) and supernatant snap-frozen in liquid nitrogen. Before SDS–PAGE analysis, proteins were concentrated and precipitated in 25% Trichloroacetic acid (TCA). Pellets were washed once in 10% TCA and three times in deionized water. Proteins were resuspended in lysis buffer as stated above whilst heating pellet at 80 °C until complete resuspension. An amount of 80 μg of protein were resolved onto 10% acrylamide gels. ECL protein detection (Millipore) was performed with a Fusion Fx7 chemiluminescent imager and the quantifications of the amounts of proteins of interest were determined by densitometry using Image J software (NIH) and normalized to respective non-phosphorylated proteins or to total protein stained with Amido black/Ponceau Red.

### Immunohistochemistry

Formalin-fixed, paraffin-embedded, 5-μm-thick human or mouse PDAC sections were deparaffinized in xylene and rehydrated through a series of graded ethanol concentrations. Antigen retrieval was performed in citrate buffer pH6 (Diapath)/Tween 0.05% or target retrieval solution at pH 6 or 9 (Dako), before quenching endogenous peroxidase activity (3% (vol/vol) H_2_O_2_). Tissue sections were then incubated with the following primary antibodies: P4HA3 (1:100, Abcam, #ab101657), Prolidase (1:50, Novus biological, #H00005184-M01), PRODH1 (1:100, Sigma-Aldrich, #SAB2501795), Collagen Ia1 (1:100, Abcam, #ab34710), Collagen IV (1:200, Abcam, #ab6586), uPARAP/Endo180 (1:100, Abcam, #ab70132), or Ki67 (1:100, BioLegend, 652402). Immunoreactivity was visualized using the Vectastain ABC kit (PK-4001, Vector Laboratories) or with biotinylated goat anti-rabbit antibody and peroxydase-conjugated streptavidin from Dako (France). Peroxydase activity was revealed using liquid DAB^+^ substrate chromogen system (Dako). Counter-staining in Mayer’s Haematoxilin was followed by a bluing step in sodium bicarbonate buffer (0.1% in water) before final dehydration and mounting of the sections. For collagen staining, Masson’s trichrome coloration was performed using an Artisan Link Special Staining System (Dako), according to manufacturer’s instructions. Positive PRODH1 areas into PDAC and normal pancreases from patients were determined from × 20 magnification images using Image J software (NIH). Quantitation of collagen staining was performed using ImageJ software (NIH).

### Immunofluorescence

Paraffin-embedded mice PDAC sections (5 μm) were treated as for IHC before blocking in PBS/3% BSA (GE Healthcare). Tissue sections were then incubated with primary antibodies, followed by incubation with Alexa fluor 568- or Alexa fluor 488-conjugated secondary antibodies (1:500, Molecular Probes). Primary antibodies were: wide-spectrum cytokeratin (1:50, wsKRT, Acris Antibodies #BP5069) or the same ones used for IHC (P4HA3, PRODH1, prolidase). Stained tissue sections were mounted using Prolong Gold Antifade reagent with DAPI (Life Technologies). For immuno-cytofluorescence, 1.5 × 10^5^ PK4A cells were seeded on 60-μm-thick glass coverslips in complete medium. Twenty-four hours later plates were washed in PBS, and experimental media added, supplemented with DQ-Fluorescein-conjugated collagen IV (20 μg ml^−1^, Life technologies) with or without EIPA (5-N-ethyl N-isopropyl amiloride (EIPA), 30 μM in DMSO), either under normoxia (21% oxygen) or hypoxia (1% oxygen). Cysteine protease inhibitor E64 (20 μM in water) was added to each condition to slow collagen lysosomal degradation, thus promotes its accumulation and visualization as described^53^. The day of the experiment, cells were washed in PBS, and fixed in cold acetone. For LAMP1 staining the primary antibody LAMP1 (1:50, Santa Cruz Biotechnology, #sc19992) was used followed by incubation with Alexa fluor 568-conjugated secondary antibodies (1:500, Molecular Probes). Coverslips were rinsed in PBS, and directly mounted using Prolong Gold Antifade reagent with DAPI (Life Technologies). Photographs were taken using a LSM 780 Confocal Inverted Microscope (Zeiss) at × 40 or × 63 magnification (under immersion).

### Collagen uptake experiment

A total of 1.5 × 10^5^ PK4A cells were seeded on 12-well plates in duplicates and cultured 48 h in complete, glucose-deprived or glutamine-deprived DMEM, with or without DQ-Fluorescein-conjugated collagen IV or I (20 μg ml^−1^, Life Technologies). Cysteine proteases inhibitor E64 (20 μM) was added to each condition as described above. EIPA was used at 30 μM. 48 h later, duplicates were pooled, cells washed in PBS and trypsinized (37 °C), pelleted, resuspended in bacterial collagenase (1 mg ml^−1^, 5 min, room temperature (RT)) and quenched in Trypan Blue to eliminate external fluorescence (7 min, RT), as described in Ikenaga *et al*.^33^ and analysed by fluorescence-activated cell sorting (FacsCalibur, BD Biosciences). Experiments were conducted in parallel in normoxia (21%) and hypoxia (1% oxygen using a Whitley Hypoxic WorkStation, Don Whitley Scientific).

### Establishment of sg-PRODH1-PK4A cell lines

A total of 0.6 × 10^5^ PK4A cells were seeded on six-well plates 24 h before transfection with a pool of three PRODH Crispr/Cas9 KO plasmids (Santa Cruz Biotechnologies, #sc-422425) each encoding the Cas9 nuclease, a Green Fluorescent Protein (GFP) and a target-specific 20 nt guide RNA (gRNA, sc-422425A1: sense: 5′-TATCAGGTGCACCCCGCCTT-3′, sc-422425A2: sense: 5′-AGTCTATCAGGCCTCTGATC-3′ sc-422425A3: sense: 5′-AGCCATTAAGCTCACTGCAC-3′), using Lipofectamine 3000 (Life Technologies). 48 h post transfection, green fluorescent cells were cloned by cell sorting using the fluorescence-activated cell sorting (ArialII, BD Biosciences). Clones were then selected for maximal PRODH1 extinction.

### Survival and proliferation assays

For survival assay, the experiments were performed by culturing 1.2 × 10^5^ PK4A cells in 24-well plates under 21% of oxygen (normoxia) or 1% oxygen (hypoxia) either in complete medium (25 mM glucose, 4 mM glutamine DMEM) or in media with low glucose (0 mM, 1 mM or 5 mM glucose DMEM) or low glutamine (0 mM or 0.5 mM glutamine DMEM) alone or supplemented with either DHP (1 mM) or with DHP and L-proline (500 μM). Each condition was repeated in quadruplicate. After 24 h, quadruplicates for each condition were pooled, and viability assessed via Trypan blue exclusion using a cell viability analyser (Vi-cell XR, Beckman Coulter). For proliferation assay, cells were plated and cultured 24–96 h in same conditions as survival assay, except that media were replenished every day. At each time point, cell viability was assessed via Trypan blue exclusion using a cell viability analyser (Vi-cell XR, Beckman Coulter). Survival assay under soluble collagen treatment were performed by culturing 4 × 10^6^ PK4A cells on 10 cm petri dishes in no glucose DMEM with or without water-soluble collagen IV (20 μg ml^−1^), EIPA (30 μM) or both compounds. After 72 h, media were replaced by complete DMEM for 24 h. Cells were fixed and stained with crystal violet (0.05% in methanol). Crystal violet was then dissolved in acetic acid (10% in water), and staining quantified by spectrophotometry at 590 nm. For cell survival experiments using collagen I-coated, 5 × 10^5^ PK4A cells were cultured 96 h on collagen I-coated versus uncoated six-well plates, with no glucose DMEM. Cells were further fixed and coloured with crystal violet (0.05% in methanol) and representative photographs were taken.

### Clonogenic assays

For clonogenic assays, 5 × 10^2^ Sg-Control and Sg-PRODH1_clone9 and _clone3 PK4a cells were seeded into a six-well plate and cultured in complete media, 5 or 1 mM glucose DMEM media. Ten days later, colonies were fixed in 10% (wt/vol) formalin, stained with 0.5% Crystal violet, imaged and quantified by ImageJ software (NIH).

### PDAC syngeneic graft models

Under isoflurane anaesthesia (induction: 4% (vol/vol) and maintenance: 1.5% (vol/vol)), 5-week-old male Naval Medical Research Institute (NMRI) nude mice (Envigo, France) were subcutaneously implanted between the shoulders with 2 × 10^6^ Sg-Control or Sg-PRODH1_clone 9 or _clone 3 PK4a cells. At the time of sacrifice (15 days post injection), tumours were extracted from the mice and sizes were determined with calipers. Tumour volume was established with the following formula: *π*/6 × [(*L*+*W*)/2].

### Nutrient uptake kinetics

Glucose and glutamine consumption or lactate production were assessed using the YSI 2950 BioAnalyser (System-C-Industry). Briefly, quadruplicates of subconfluent PK4A cells were cultured in 48-well plates over 24 h either in 25 mM glucose, 4 mM glutamine DMEM, low glutamine (2, 0.5 mM), no glutamine or low glucose (5, 1 mM), no glucose media. At each end point, supernatants were collected and metabolite concentrations measured. For glucose uptake and lactate production assessment under impairment of proline degradation, 1.2 × 10^5^ PK4A cells were seeded in quadruplicates in 24-well plates in 0.5 mM glutamine DMEM alone or supplemented with DHP (1 mM), L-proline (500 μM) or both compounds. After 24 h, supernatants were collected and glucose and lactate concentrations measured as above. Viable cells numbers were evaluated through Trypan blue exclusion test using a semi-automatic cell counter (Countess, Life Technologies) and used for normalization. For every metabolite measurements, fresh complete or nutrient-deprived media were used as starting point for baseline concentrations.

### Tracing experiments

For free proline tracing under normoxia, 1.5 × 10^5^ PK4A cells were plated in triplicate in six-well plates in complete medium. The following day, cells were washed in PBS and refreshed with DMEM without pyruvate, supplemented with 10% dialysed FBS and 500 μM U-^13^C-proline for 24 h. ECM with U-^13^C-proline was prepared according to methods adapted from Vlodavsky *et al*.^54^. Briefly, primary MEFs were seeded at 75 × 10^3^ cells/six-well on fibronectin pre-coated plates (BD Biosciences) in DMEM (with pyruvate) with 10% FBS. One day after plating, cells were switched into DMEM (with pyruvate) with 10% dialysed FBS supplemented with 500 μM U-^13^C-proline and 100 μM Vitamin C. Cells were cultured for 6 days in this media, with media renewal every 48 h. After six days, MEFs were removed by washing in 1 ml per well PBS with 0.5% (v/v) Triton X-100 and 20 mM NH_4_OH. Labelled ECM was washed four times with PBS before plating 1.5 × 10^5^ PK4A cells per well. The following day, PK4A cells were switched into the indicated media (with 0.5% dialysed FBS) for 24 h. For free proline tracing experiments in hypoxia, 1 × 10^5^ cells were plated 6 h before initiation of the experiment. At the start of the experiment, cells were washed once with PBS and then switched into the indicated experimental media containing 500 μM U-^13^C-proline and placed for 24 h under normoxic (21% oxygen) or hypoxic (1% oxygen) conditions. After 24 h, cells were harvested, extracted and analysed as described below. For glucose tracings to lactate, 5 × 10^4^ cells were plated 1 day before initiation of the experiment. At the start of the experiment, cells were washed once with PBS and then switched into the indicated experimental media containing 25 mM U-^13^C-glucose with 4 mM, 2 mM or 0.5 mM glutamine. At the indicated time points, cells were harvested, extracted and analysed as described below. Tracing under DHP was similar apart from the following exceptions: 1 × 10^5^ cells were plated 6 h before initiation of the experiment. At the start of the experiment, cells were washed once with PBS and then switched into the indicated experimental media containing 500 μM U-^13^C-proline±1 mM DHP. After 24 h, cells were harvested, extracted and analysed as described below. In experiments where normalization to cell number was needed, a parallel plate maintained under the same condition was used and tracing results were normalized to raw cell counts (via microscopy) from those plates.

### Extraction of metabolites

At the conclusion of tracing experiments, cells were rinsed once in water and snap-frozen in liquid nitrogen and metabolites were extracted with a methanol:chloroform:water (5:5:3) mix, as previously described^55^. After vortexing and centrifugation to clarify, the aqueous phase containing polar metabolites was removed, dried down under nitrogen gas and stored at −80 °C for subsequent derivatization. Acid hydrolysis was performed on U-^13^C-proline labelled ECM to assess fractional incorporation of proline according to the protocol adapted from Antoniewicz *et al*.^57^. Briefly, ECM was boiled in 500 μl 18% hydrochloric acid O/N at 100 °C. Two-fifty microlitre supernatant was evaporated under nitrogen and frozen at −80 °C for subsequent derivatization. To determine the relative abundance of amino acids in *in vitro*-generated ECM, we adapted a previously described^41^ protocol. Briefly, the samples were boiled at 100 °C overnight in 18% hydrochloric acid modified to include an amino acid internal standard mixture of U-^13^C and U-^15^N species (Cambridge Isotopes, # MSK-A2-1.2) at a final concentration of 100 μM. Samples were then run on the gas chromatography-mass spectrometry as described in the following section. The ratio of ion counts of unlabelled and fully labelled versions of each amino acid was calculated. The fully labelled standards contain each amino acid at equal concentrations, so these calculated ratios represent their relative abundances. We therefore normalized the sum of these ratios to equal one to calculate the fraction of protein amino acids represented by each individual amino acid. Cysteine and tryptophan residues are acid labile and not recoverable from the hydrolyzed samples. Histidine was also not recovered from the samples. Fractionation using trizol and determination of relative contributions were conducted as previously described^34^. Unique to this experiment, cells were cultured for 72 h with daily refreshing in indicated media conditions supplemented 500 μM proline with tracer of 0.05 μCi ml^−1^ [U-^14^C]-proline.

### Gas chromatography/mass spectrometry analyses

Dried polar metabolites were dissolved in 20 μl of 2% methoxyamine hydrochloride in pyridine (ThermoFischer Scientific) and held at 37 °C for 1.5 h. After dissolution and reaction, tert-butyl-di-methyl-silyl derivatization was initiated by adding 25 μl *N*-methyl-*N*-(tert-butyl-di-methyl-silyl) trifluoroacetamide+1% tert-butyl-dimethyl-chlorosilane (Sigma-Aldrich) and incubating at 37 °C for 1hr. For acid hydrolysates, 20 μl pyridine (Sigma-Aldrich) was used in place of 2% methoxyamine hydrochloride in pyridine. 25 μl *N*-methyl-*N*-(tert-butyl-di-methyl-silyl) trifluoroacetamide+1% tert-butyl-dimethyl-chlorosilane and incubating at 37 °C for 1hr was performed as with polar metabolites. GC/MS analysis was performed using an Agilent 7890 GC equipped with a 30 m DB-35MS capillary column connected to an Agilent 5975B MS operating under electron impact ionization at 70 eV. One microliter of sample was injected in splitless mode at 270 °C, using helium as the carrier gas at a flow rate of 1 ml min^−1^. For measurement of amino acids, the GC oven temperature was held at 100 °C for 3 min and increased to 300 °C at 3.5 °C min^−1^. The MS source and quadrupole were held at 230 °C and 150 °C, respectively, and the detector was run in scanning mode, recording ion abundance in the range of 100–605 *m*/*z*. MIDs were determined by integrating the appropriate ion fragments listed in supplementary Table 2 and corrected for natural isotope abundance using an algorithm adapted from Fernandez and colleagues^58^. MIDs are listed in Supplementary Data 1.

### Mitochondrial oxygen consumption

Oxygen consumption rates were determined using a Seahorse XF24 extracellular flux analyser (Seahorse Bioscience, Billerica, MA, USA). PK4A cells were seeded in XF24 V7 multi-well plates (30,000 cells per well). The next day, cells were treated with DHP (1 mM) alone or combined with L-Proline (0.5 mM) for 24 h in media containing 0.5 mM glutamine, 5 mM or 1 mM glucose. The day of measurements, culture media was replaced with assay media (DMEM without phenol red, 143 mM NaCl, 100 mM sodium pyruvate with 0.5 mM glutamine, 5 mM or 1 mM glucose, pH7.4) and plates incubated at 37 °C for 1hr before the start of measurement. OCR (oxygen consumption rate) was measured before and after sequential injection of the following inhibitors: oligomycin 1 μM, FCCP 1 μM and rotenone/antimycin 0.5 μM. OCR were normalized to viable PK4A cells determined by Trypan blue exclusion test using a semi-automatic cell counter (Countess, Life technologies).

### Statistical analysis

Two-tailed unpaired Student’s *t*-test was used for comparing means of two experimental groups in the case of one independent factor, except for PDAC (*n*=10) and normal pancreases (*n*=11) PRODH1 levels comparison, we used a Mann–Whitney non-parametric test since data do not follow a normal distribution. Two-way analysis of variance, followed by the Bonferroni test for multiple comparisons, was used when comparing more than two experimental groups (Prism 6 software, version 6.01). In all figures, data are presented as mean±s.e.m. and exact sample size (*n*), corresponding to biological replicates, used to calculate the statistics are indicated. *P*<0.05 values were considered statistically significant. No statistical methods were used to predetermine sample size. Raw data are available in Supplementary Fig. 10a,b.

### Data availability

The authors declare that the data supporting the findings of this study are available within the paper and its Supplementary Information files or from the corresponding author upon reasonable request.

## Additional information

**How to cite this article:** Olivares, O. *et al*. Collagen-derived proline promotes pancreatic ductal adenocarcinoma cell survival under nutrient limited conditions. *Nat. Commun.* **8,** 16031 doi: 10.1038/ncomms16031 (2017).

**Publisher’s note:** Springer Nature remains neutral with regard to jurisdictional claims in published maps and institutional affiliations.

## Supplementary Material

Supplementary Information

Supplementary Data 1
