# Supplementary material for: Collagen-derived proline promotes pancreatic ductal adenocarcinoma cell survival under nutrient limited conditions
Source: Nat Commun. 2017 Jul 7;8:16031. doi: 10.1038/ncomms16031 (PMC5504351; doi:10.1038/ncomms16031)
Supplement: Supplementary Information [file ncomms16031-s1.pdf]

Title of file for HTML: Supplementary Information
Description: Supplementary Figures and Supplementary Tables

Title of file for HTML: Supplementary Data 1
Description: Complete mass isotopomer distribution of data displayed in Fig 6d and Fig S7d from U-13C-proline after 24hrs. All data are presented as mean ± SEM. n=3 replicates per condition. M0 represents unlabelled molecules with each increasing number representing additional 13C-labelled carbons.

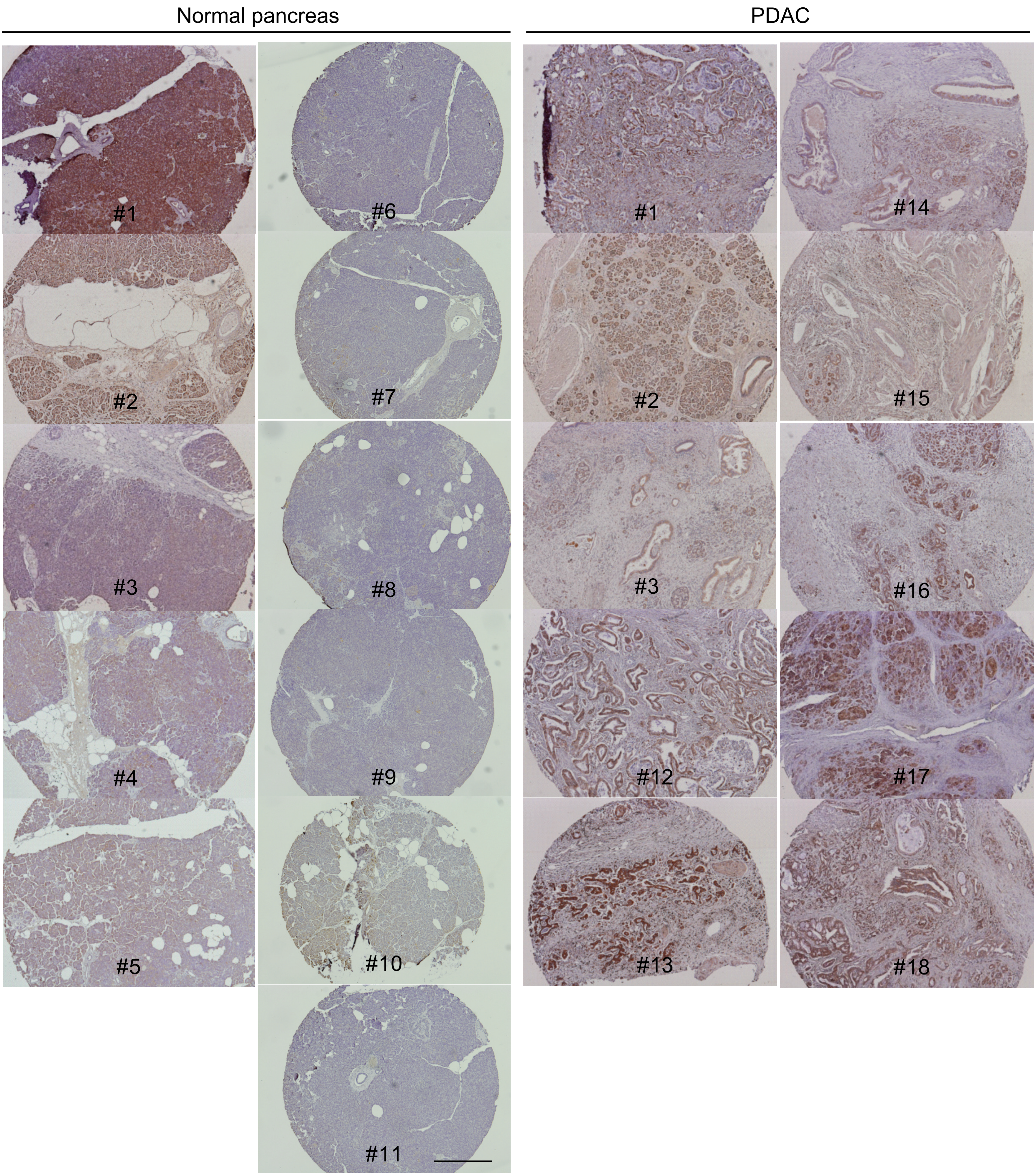

Supplementary Figure 1

PRODH1 is expressed in human PDAC samples. Staining of PRODH1 by IHC on human tissue macroarray (TMA) containing PDAC (n=10), adjacent normal pancreas (n=3, #1 to #3) and normal pancreas from PDAC-free patients (n=8, #4 to #11). Quantification of PRODH1 expression in TMA samples was determined by densitometry using Image J software (NIH) with the same threshold for all images. Scale bar: 500 µm.

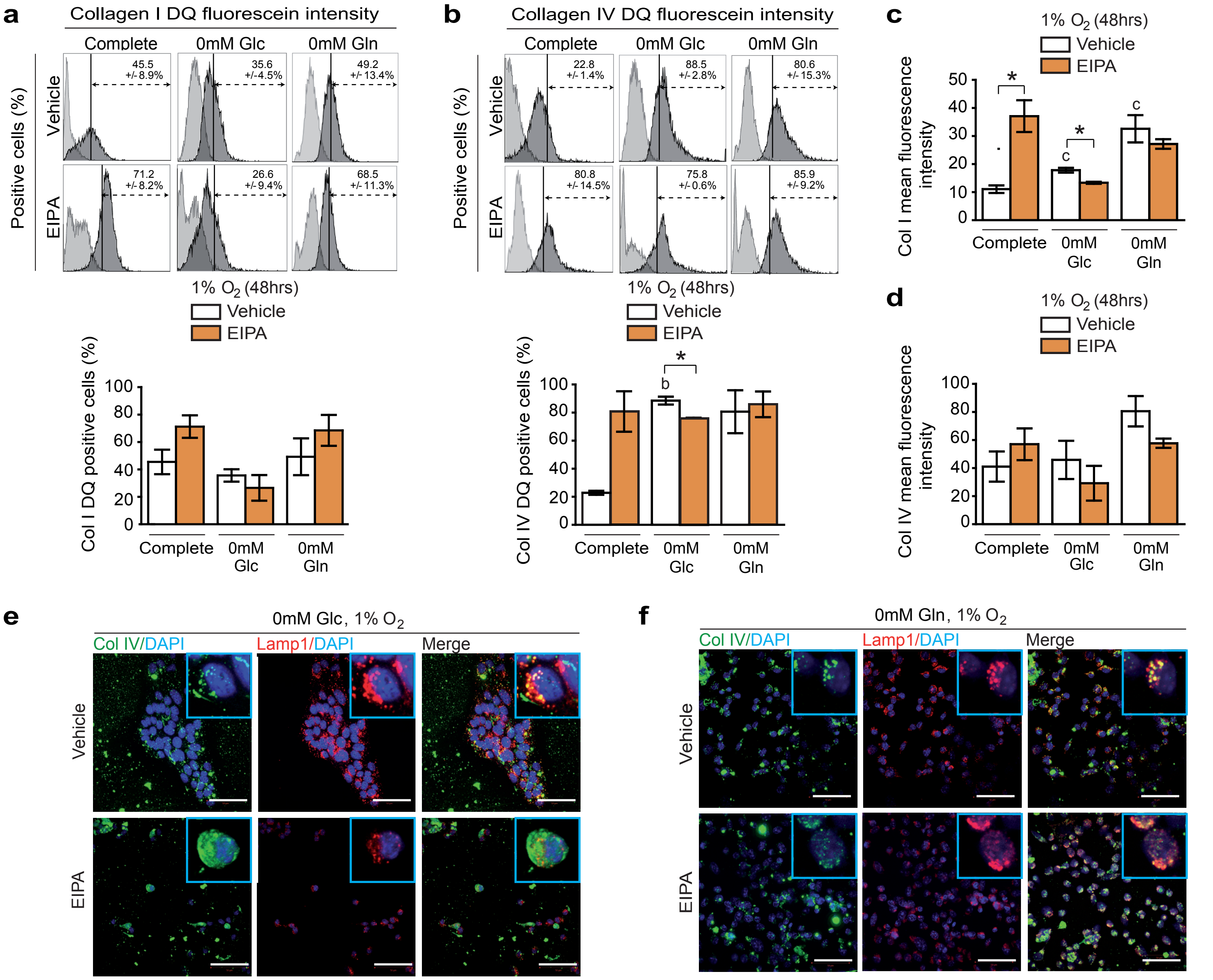

Supplementary Figure 2

PDAC cells take up and degrade collagen after glucose or glutamine deprivation under hypoxia. (**a**) and (**b**) Representative flow cytometry profiles (upper panels) and charts (lower panels) representing the percentage of PK4A cells taking up collagen I (**a**) or IV (**b**) DQ. Mean (**c**) Col I or (**d**) Col IV DQ fluorescence intensity (MFI) in PK4A cells cultured during 48hrs under hypoxia (1% O2) as in Fig. 3a-b. Data represent mean ± SEM (n=3 independent experiments). P values, indicated by asterisks, show statistical significance relative to respective culture condition without EIPA (* p<0.05), while letters indicate statistical significance relative to complete media without EIPA ([c] p<0.05, [b] p<0.01). Two-tailed unpaired Student's t-test. (**e**) and (**f**) Representative co-staining of Col IV DQ and Lamp1 lysosomal marker in PK4A cells cultured in glucose-free (**e**) or glutamine-free (**f**) media under hypoxic conditions with or without EIPA. Insets show magnification of Col IV, Lamp1 or Col IV/Lamp1 (merge) PKA4 cells in each condition. Images are representative of n=3 independent experiments. Scale bar: 50 µm.

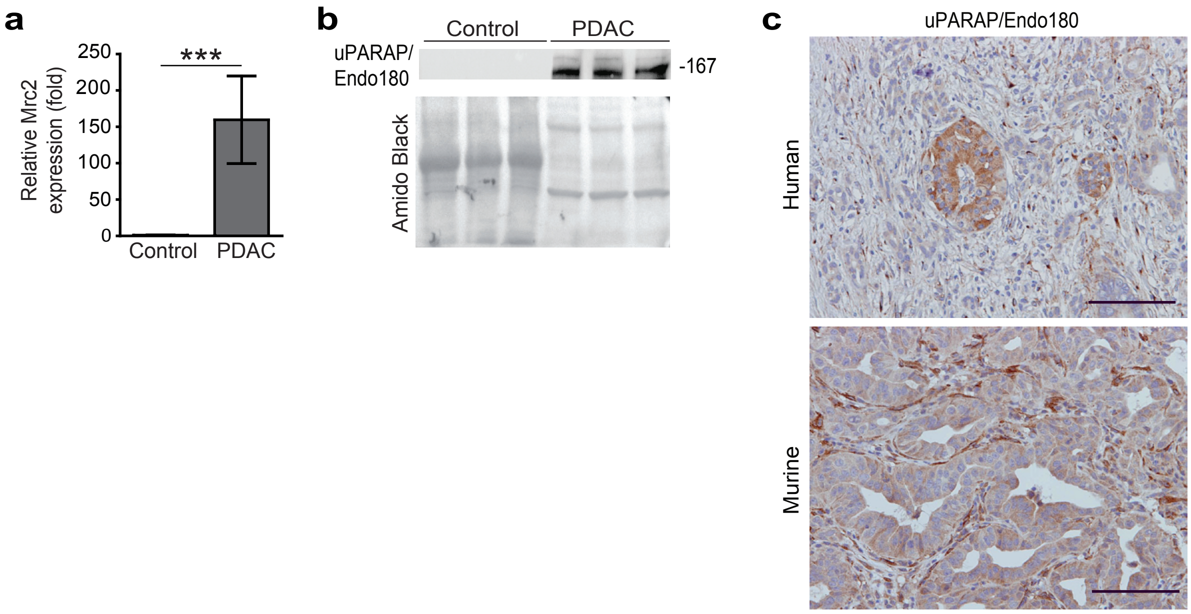

Supplementary Figure 3

MRC2/Endo180 is expressed by PDAC tumour cells. (**a**) mRNA levels of the receptor uPARAP/Endo180 (Mrc2 gene) measured by quantitative RT-PCR in PDAC from PKI mice (n=3) versus control pancreases from KI mice (n=3). Data are presented as mean ± SEM. *** p<0.001; two-tailed unpaired Student's t-test. (**b**) Protein levels of uPARAP/Endo180 in PDAC and control pancreas from PKI and KI mice, respectively (upper panel). Marker size in kDa is indicated. Total protein stained with Amido black were used as loading control (lower panel). Uncropped images of blots are shown in Supplementary Fig. 10. (**c**) Representative uPARAP/Endo180 staining in human and murine PDAC sections (n=4 patients and n=3 PKI mice). Scale bar: 100 μm.

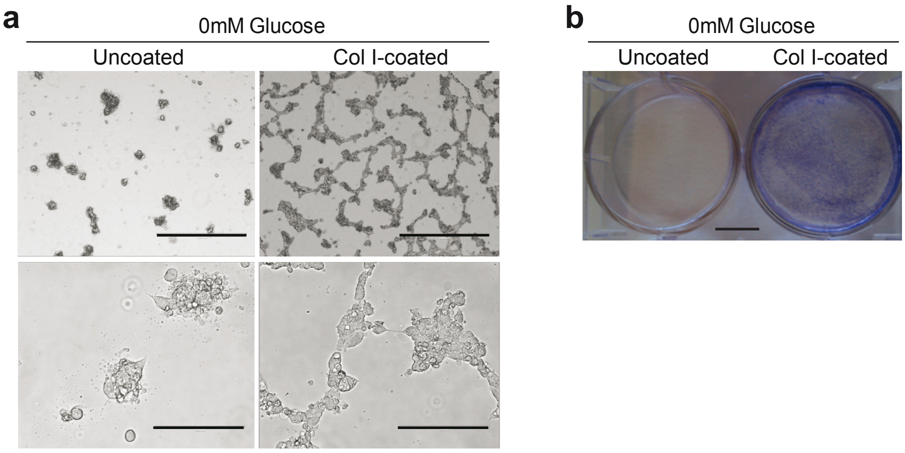

Supplementary Figure 4

Collagen promotes PDAC cell survival. (**a**) and (**b**) Survival of PK4A cells cultured in glucose-free media on uncoated or coated-collagen I plates. Representative images of unstained cells (**a**) and cells stained with crystal violet (**b**) after 96hrs of treatment (n=2 independent experiments). Scale bar: 1,000 μm (**a**: upper images), 200 μm (**a**: lower images) and 5 mm (**b**).

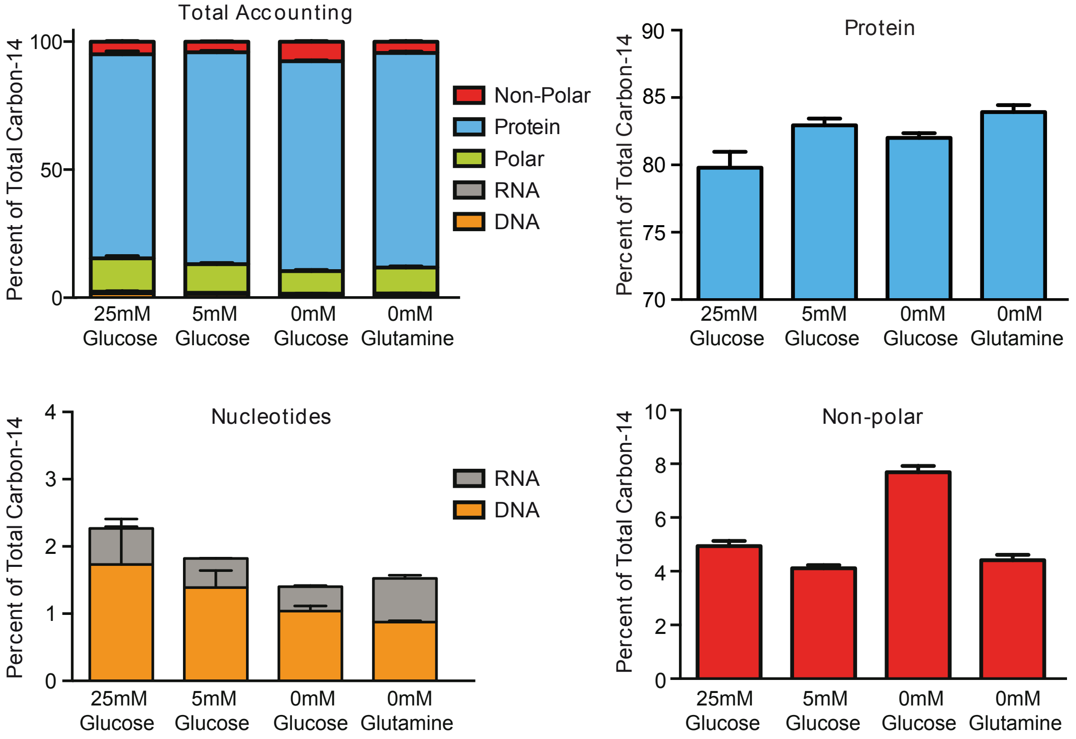

Supplementary Figure 5

Accounting of proline-derived carbon contributions to different cellular macromolecules. Relative contributions of proline to proteins, nucleotides (RNA, DNA) and non-polar (lipids) fractions determined under different nutrient conditions. Each bar represents the average of n=3 biological replicates ± SEM.

a

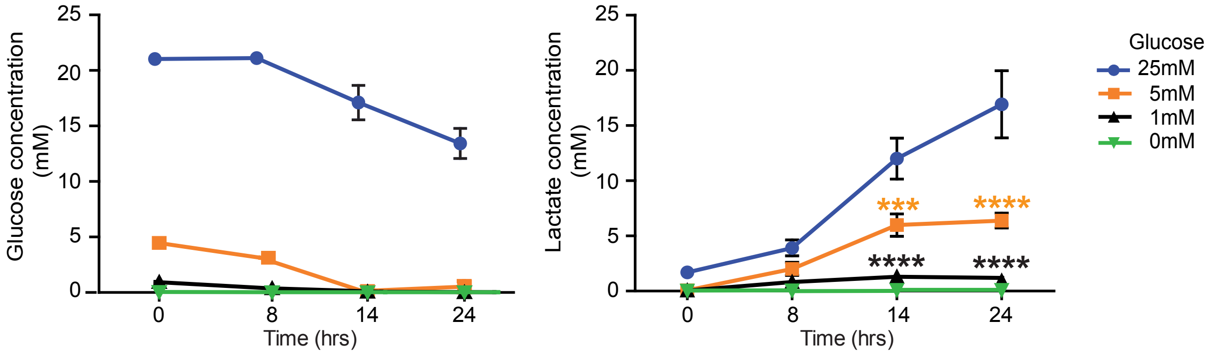

b

Time	1hr			3hrs			6hrs			24hrs		
**Glutamine**	**4mM**	**2mM**	**0.5mM**	**4mM**	**2mM**	**0.5mM**	**4mM**	**2mM**	**0.5mM**	**4mM**	**2mM**	**0.5mM**
**M0**	0.1694	0.1822	0.2179	0.1440	0.1487	0.1440	0.1015	0.1024	0.1221	0.0399	0.0357	0.0457
SEM	0.0099	0.0041	0.0416	0.0148	0.0110	0.0145	0.0163	0.0082	0.0058	0.0007	0.0014	0.0021
**M1**	0.0019	0.0015	0.0022	0.0022	0.0027	0.0026	0.0032	0.0027	0.0032	0.0042	0.0041	0.0033
SEM	0.0004	0.0007	0.0006	0.0006	0.0004	0.0001	0.0003	0.0003	0.0001	0.0001	0.0001	0.0001
**M2**	0.0233	0.0227	0.0220	0.0248	0.0237	0.0244	0.0264	0.0262	0.0259	0.0296	0.0296	0.0278
SEM	0.0004	0.0011	0.0011	0.0006	0.0003	0.0007	0.0007	0.0009	0.0005	0.0003	0.0001	0.0003
**M3**	0.8092	0.7972	0.7603	0.8315	0.8288	0.8317	0.8717	0.8724	0.8532	0.9283	0.9327	0.9269
SEM	0.0094	0.0036	0.0398	0.0142	0.0104	0.0149	0.0155	0.0073	0.0055	0.0004	0.0017	0.0023

Supplementary Figure 6

Glutamine depletion does not change relative glucose contribution to lactate production. (**a**) Glucose uptake (left panel) and lactate production (right panel) by subconfluent PK4A cells cultured under gradual glucose deprivation (25mM, 5mM, 1mM and 0mM) at 8, 14 and 24hrs. Data are mean ± SEM, n=4 independent experiments. P value indicated by asterisks is relative to lactate value in 25mM glucose media at each time point. *** p< 0.001, **** p< 0.0001; two-way ANOVA followed by Bonferroni post-hoc test. (**b**) Complete mass isotopomer distribution (MID) of lactate from U-13C-glucose at 1, 3, 6 and 24hrs under a gradual decline in glutamine concentration. All data are presented as mean ± SEM. n=3 replicates per condition.

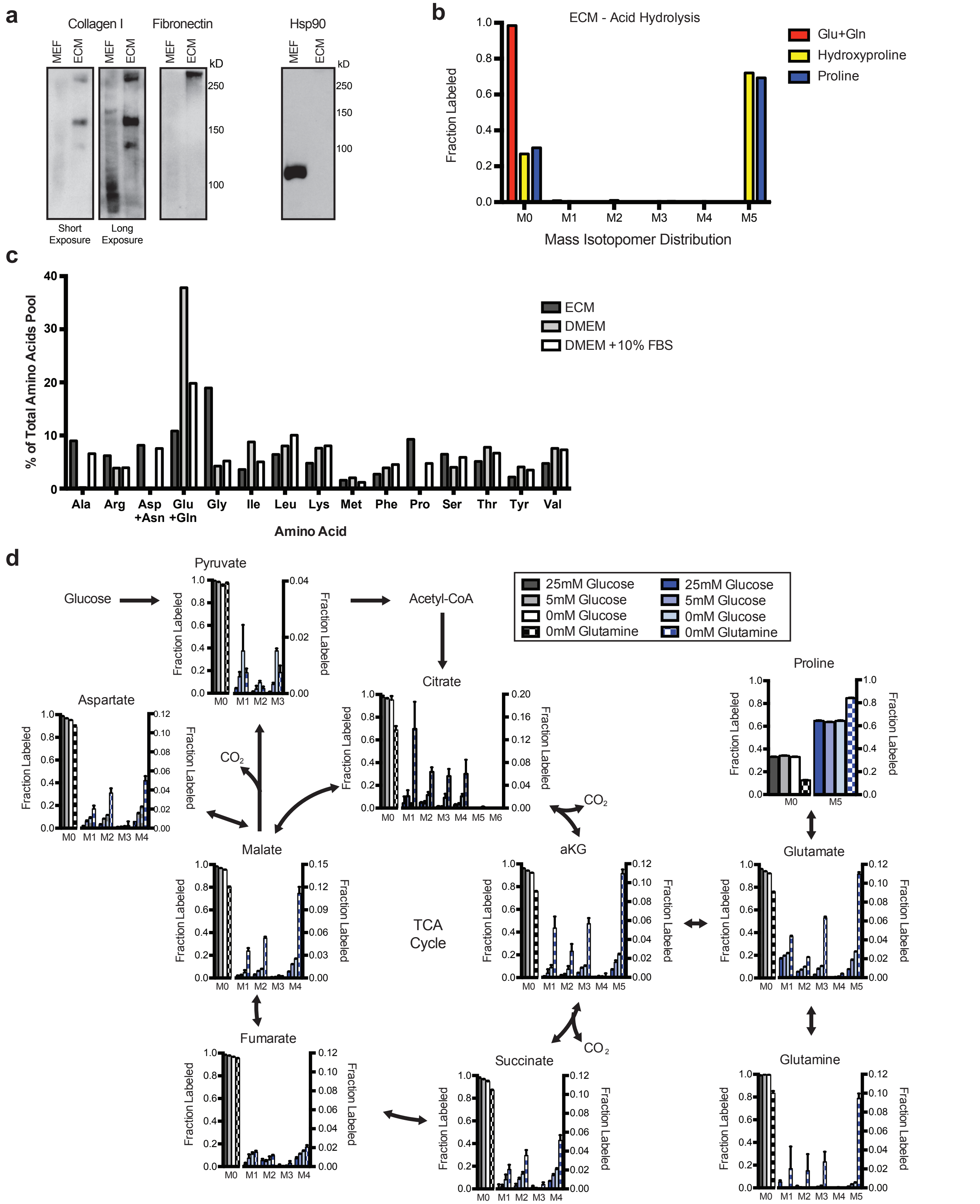

Supplementary Figure 7

ECM is proline-rich and essential amino acid poor and proline supplies the TCA cycle. (**a**) Western blot of primary MEFs and de-cellularised ECM indicating successful de-cellularisation of ECM prior to PK4A cells plating. Uncropped blots are representative of n=2 independent experiments. (**b**) Full mass isotopomer distribution of labelled ECM after acid hydrolysis. Data is from n=1 experiment and is representative of n=2 experiments. M0 represents unlabelled molecules with each increasing number representing additional ^{13}C-labelled carbons. (**c**) Percent of total protein pool of each amino acid following acid hydrolysis of fibroblast-synthesized ECM, DMEM and DMEM + 10% FBS. (**d**) Full GCMS tracing analysis of U-^{13}C-proline into central carbon metabolism in PDAC cells after 24hrs under indicated nutrient conditions. M0 represents unlabelled molecules with each increasing number representing another labelled carbon.

a

Proline

Time	25mM Glucose + 4mM Gln		5mM Glucose + 4mM Gln		0mM Glucose + 4mM Gln		25mM Glucose + 0mM Gln	
O_2	21%	1%	21%	1%	21%	1%	21%	1%
**M0**	0.0850	0.1014	0.1292	0.1671	0.1213	0.0947	0.0284	0.0294
SEM	0.0024	0.0014	0.0014	0.0027	0.0017	0.0023	0.0006	0.0007
**M1**	-0.0018	-0.0023	-0.0030	-0.0039	-0.0019	-0.0017	-0.0001	-0.0003
SEM	0.0002	0.0000	0.0002	0.0002	0.0001	0.0001	0.0001	0.0001
**M2**	0.0007	0.0008	0.0009	0.0007	0.0012	0.0006	0.0005	0.0004
SEM	0.0001	0.0000	0.0001	0.0001	0.0000	0.0002	0.0001	0.0000
**M3**	0.0056	0.0053	0.0055	0.0051	0.0062	0.0057	0.0059	0.0060
SEM	0.0000	0.0001	0.0001	0.0000	0.0002	0.0001	0.0001	0.0000
**M4**	0.0406	0.0396	0.0386	0.0368	0.0388	0.0397	0.0429	0.0424
SEM	0.0002	0.0002	0.0002	0.0002	0.0002	0.0003	0.0000	0.0003
**M5**	0.8845	0.8707	0.8435	0.8092	0.8503	0.8766	0.9401	0.9389
SEM	0.0019	0.0011	0.0012	0.0025	0.0023	0.0026	0.0010	0.0006

Glutamate

Time	25mM Glucose + 4mM Gln		5mM Glucose + 4mM Gln		0mM Glucose + 4mM Gln		25mM Glucose + 0mM Gln	
O_2	21%	1%	21%	1%	21%	1%	21%	1%
**M0**	0.9664	0.9815	0.9793	0.9916	0.9408	0.9794	0.6268	0.6386
SEM	0.0006	0.0006	0.0004	0.0000	0.0013	0.0008	0.0004	0.0043
**M1**	0.0025	-0.0010	0.0028	0.0003	0.0059	-0.0012	0.0465	0.0301
SEM	0.0005	0.0003	0.0003	0.0005	0.0002	0.0003	0.0007	0.0023
**M2**	0.0017	-0.0004	0.0007	0.0002	0.0081	0.0023	0.0361	0.0375
SEM	0.0002	0.0001	0.0003	0.0003	0.0002	0.0002	0.0010	0.0034
**M3**	0.0069	0.0013	0.0036	0.0002	0.0101	0.0034	0.0968	0.0819
SEM	0.0001	0.0001	0.0001	0.0001	0.0003	0.0002	0.0003	0.0007
**M4**	0.0010	0.0009	0.0006	0.0004	0.0016	0.0007	0.0075	0.0090
SEM	0.0000	0.0000	0.0000	0.0001	0.0001	0.0001	0.0002	0.0007
**M5**	0.0215	0.0179	0.0131	0.0073	0.0338	0.0154	0.1888	0.2064
SEM	0.0002	0.0001	0.0001	0.0002	0.0006	0.0005	0.0001	0.0010

aKG

Time	25mM Glucose + 4mM Gln		5mM Glucose + 4mM Gln		0mM Glucose + 4mM Gln		25mM Glucose + 0mM Gln	
O_2	21%	1%	21%	1%	21%	1%	21%	1%
**M0**	0.9799	0.9859	0.9887	0.9954	0.9536	0.9836	0.6832	0.6968
SEM	0.0029	0.0013	0.0016	0.0014	0.0034	0.0008	0.0030	0.0170
**M1**	0.0034	-0.0022	-0.0007	-0.0027	0.0075	0.0035	0.0449	0.0210
SEM	0.0032	0.0009	0.0023	0.0014	0.0016	0.0022	0.0055	0.0086
**M2**	0.0030	0.0012	0.0009	0.0006	0.0115	0.0042	0.0269	0.0373
SEM	0.0025	0.0007	0.0013	0.0003	0.0033	0.0017	0.0046	0.0039
**M3**	0.0029	0.0002	0.0031	0.0007	0.0064	0.0015	0.0939	0.0777
SEM	0.0005	0.0004	0.0002	0.0005	0.0012	0.0010	0.0012	0.0023
**M4**	-0.0040	0.0000	-0.0016	0.0000	-0.0038	-0.0037	-0.0149	-0.0088
SEM	0.0006	0.0002	0.0005	0.0002	0.0013	0.0003	0.0039	0.0047
**M5**	0.0194	0.0174	0.0125	0.0072	0.0326	0.0142	0.1976	0.2079
SEM	0.0006	0.0002	0.0001	0.0005	0.0013	0.0009	0.0073	0.0032

b

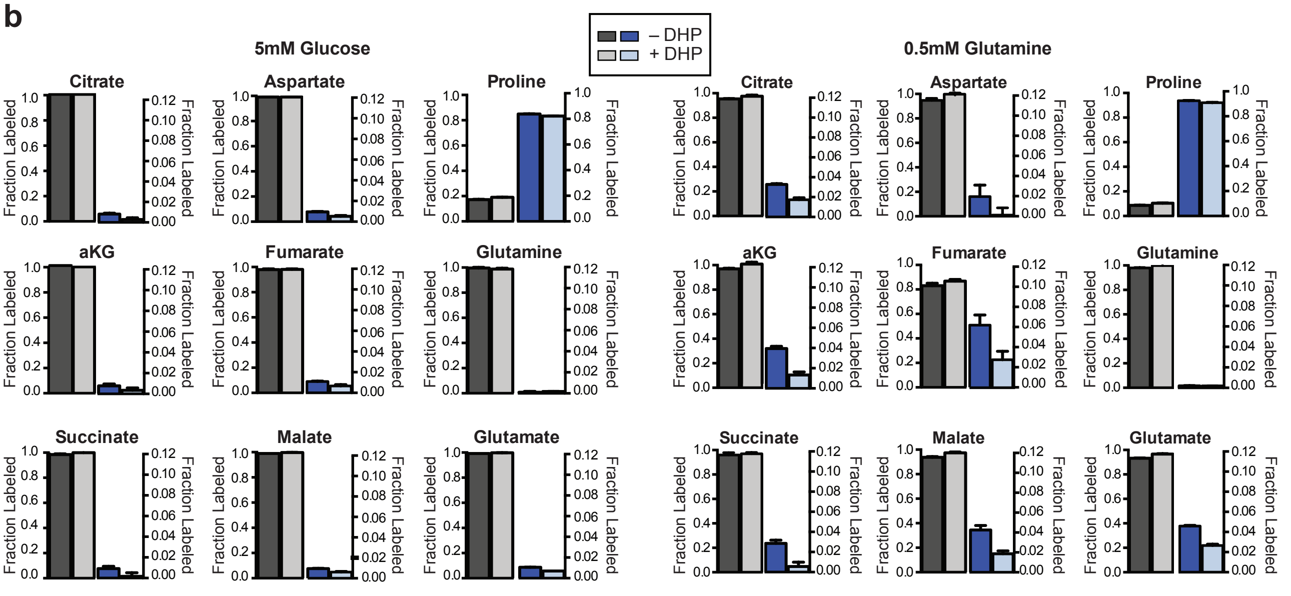

Supplementary Figure 8

Hypoxia does not influence proline conversion to glutamate or alpha-ketoglutarate but DHP inhibits metabolism of proline to TCA. (**a**) Complete mass isotopomer distribution of proline, glutamate and alpha-ketoglutarate (α-KG) from U-^{13}C-proline after 24hrs at the indicated conditions in normoxia (21% O_2) and hypoxia (1% O_2). All data are presented as mean ± SEM. n=3 replicates per condition. M0 represents un-labelled molecules with each increasing number representing additional ^{13}C-labelled carbons. (**b**) Fractional labelling of proline, glutamine and TCA intermediate from U-^{13}C-proline after 24hrs at 5mM glucose (left charts) or 0.5mM glutamine (right charts) with or without DHP 1mM. All data presented as mean ± SEM. n=3 replicates per condition.

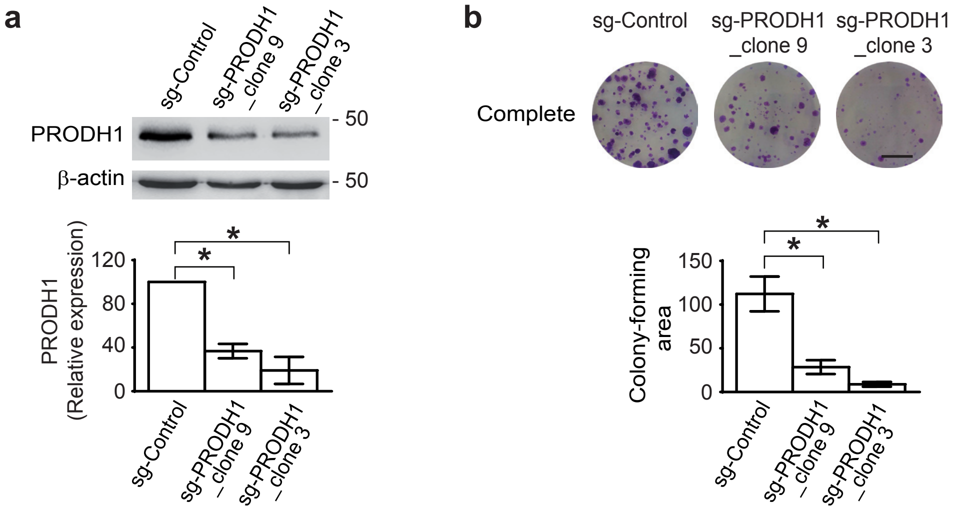

Supplementary Figure 9

Prodh1 gene-editing in PK4A cells using the CRISPR-Cas9 system. (**a**) Illustration and quantification (upper and lower panels, respectively) of PRODH1 levels in sg-Control and sg-PRODH1_clone9 and _clone3 PK4A cells revealed by western blot analysis (marker size indicated in kDa). Protein levels in each cell line are normalised to β-actin. Data are expressed as mean ± SEM (n=2 independent experiments). Uncropped images of blots are shown in supplementary Fig. 10. * $p<0.05$, two-tailed unpaired Student's t-test. (**b**) Representative clonogenic assay (upper panel) and quantification of sg-Control and sg-PRODH1_clone 9 and _clone 3 PK4A cells colony-forming area (lower panel) in complete media. Data are mean ± SEM (n=3 independent experiments). * $p<0.05$, two-tailed unpaired Student's t-test. Scale bar: 5 mm

Fig 1e

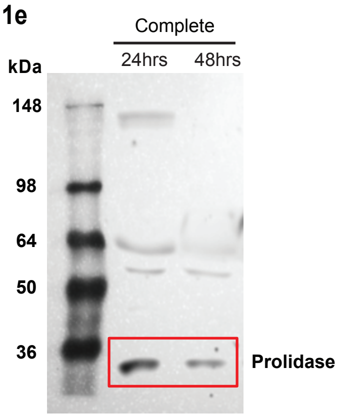

Fig 2d

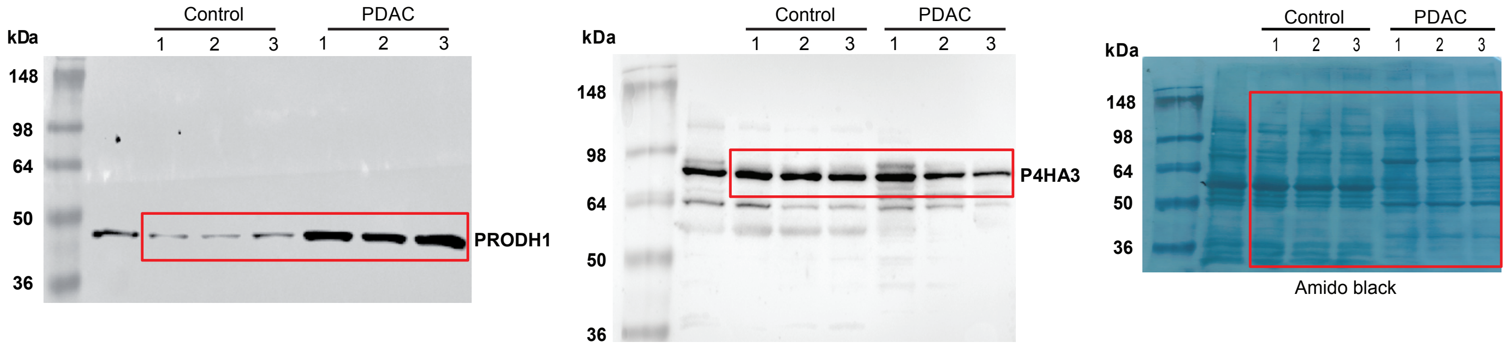

Fig 4b

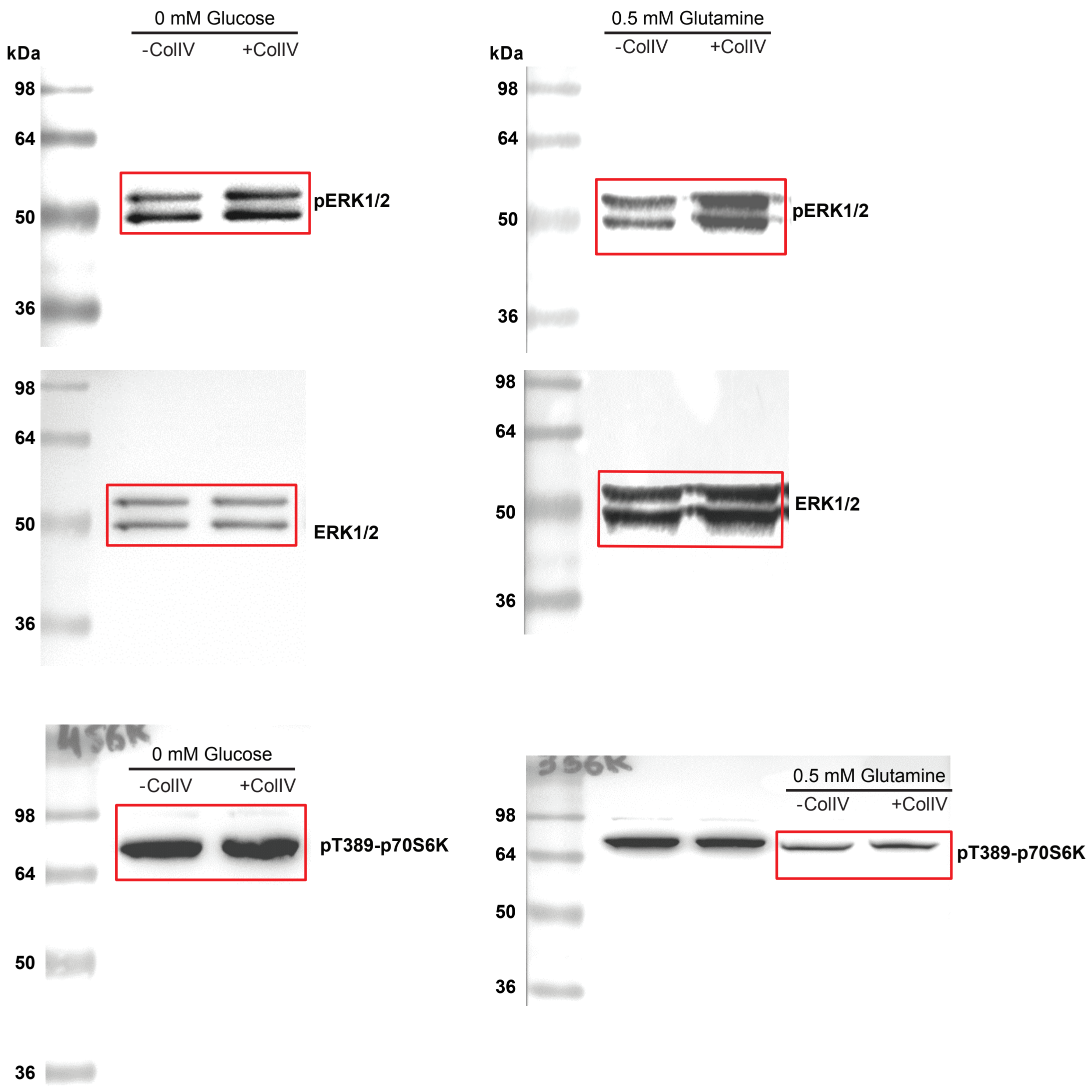

Supplementary Figure 10a

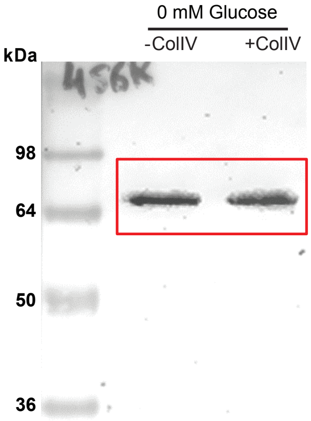

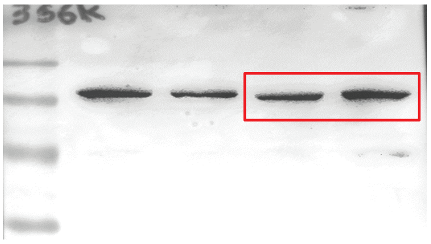

Fig 4c

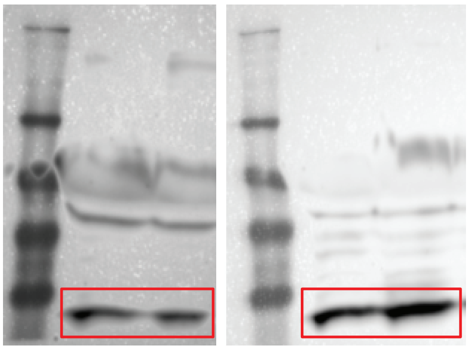

Supp. Fig 3b

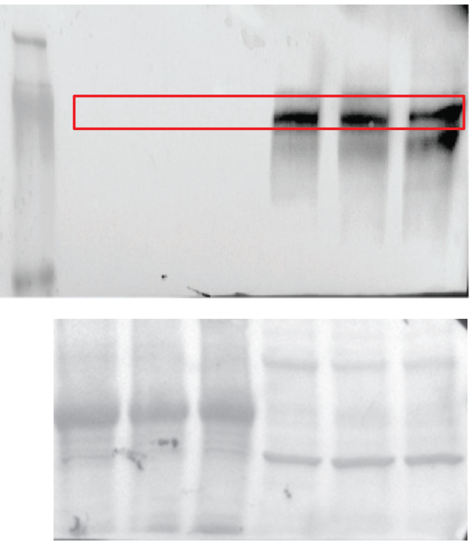

Supp. Fig 9a

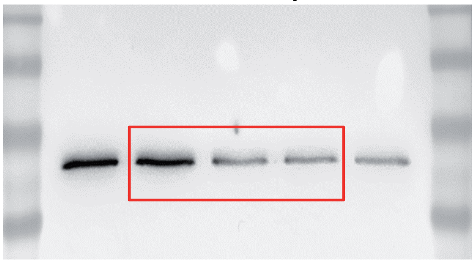

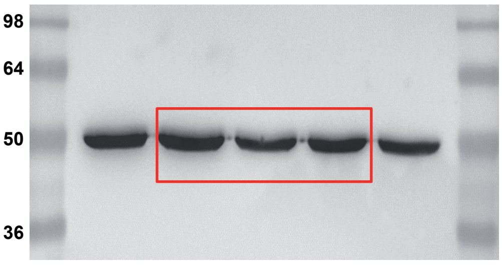

Supplementary Figure 10b

Supplementary Table 1. Primer sequences (*mus musculus*) used to determine transcript expression profiles by real-time PCR

Gene name	Forward primer (5'-->3')	Reverse primer (5'-->3')	RefSeq mRNA
pepd	GGGAAAGATCCATTCCAAGG	CACTGCCACTGTCTGTGTTG	NM_008820.2
mmp2	CCAGATCACATACAGGATCATT	CCATCATGGATTCGAGAAAA	NM_008610.2
mmp9	CAGCTGGCAGAGGCATACTT	TTCTGAAGCATCAGCAAAGC	NM_013599.3
mmp13	GGGACTAAAGAACATGGTGACT	AGCCTTTGGAACTGCTTGTC	NM_008607.2
36b4	GCTGATGGGCAAGAACACCA	CCCAAAGCCTGGAAGAAGGA	NM_007475.5
endo180	GCCCCATCAAGAGTAACGAC	CGTGATACTCAGCAAGTCTGC	NM_008626.3

Supplementary Table 2. Amino Acid Fragments Used for Isotope Quantification

Metabolite	Carbons[a]	Formula[b]	Mass (m/z)
**αKG**	12345	$C_{14}H_{28}O_5NSi_2$	346
**Ala**	23	$C_{10}H_{26}ONSi_2$	232
**Ala**	123	$C_{11}H_{26}O_2NSi_2$	260
**Arg**	23456	$C_{17}H_{38}N_3Si_2$	340
**Arg**	123456	$C_{20}H_{44}O_2N_3Si_3$	442
**Asp**	12	$C_{14}H_{32}O_2NSi_2$	302
**Asp**	234	$C_{17}H_{40}O_3NSi_3$	390
**Asp**	1234	$C_{18}H_{40}O_4NSi_3$	418
**Cit**	123456	$C_{20}H_{39}O_6Si_3$	459
**Cit**	123456	$C_{26}H_{55}O_7Si_4$	591
**Fum**	1234	$C_{12}H_{23}O_4NSi_2$	287
**Gln**	12345	$C_{19}H_{43}O_3N_2Si_3$	431
**Glu**	2345	$C_{16}H_{36}O_2NSi_2$	330
**Glu**	12345	$C_{19}H_{42}O_4NSi_3$	432
**Gly**	2	$C_9H_{24}ONSi_2$	218
**Gly**	12	$C_{10}H_{24}O_2NSi_2$	246
**Ile**	23456	$C_{11}H_{26}NSi$	200
**Ile**	23456	$C_{13}H_{32}ONSi_2$	274
**Ile**	123456	$C_{14}H_{32}O_2NSi_2$	302
**Lac**	123	$C_{11}H_{25}O_3Si_2$	261
**Leu**	23456	$C_{11}H_{26}NSi$	200
**Leu**	23456	$C_{13}H_{32}ONSi_2$	274
**Leu**	123456	$C_{14}H_{32}O_2NSi_2$	302
**Lys**	23456	$C_{17}H_{41}N_2Si_2$	329
**Lys**	123456	$C_{20}H_{47}O_2N_2Si_3$	431
**Mal**	1234	$C_{18}H_{39}O_5Si_3$	419
**Met**	2345	$C_{10}H_{24}NSiS$	218
**Met**	2345	$C_{12}H_{30}ONSi_2S$	292
**Met**	12345	$C_{13}H_{30}O_2NSi_2S$	320
**Phe**	23456789	$C_{14}H_{24}NSi$	234
**Phe**	23456789	$C_{14}H_{32}O_2NSi_2$	308
**Phe**	123456789	$C_{17}H_{30}O_2NSi_2$	336
**Pro**	12345	$C_{16}H_{36}O_2NSi_2$	330
**(Hydroxy)Pro**	12345	$C_{19}H_{45}O_3NSi_3$	416
**Pyr**	123	$C_6H_{12}O_3NSi$	174
**Suc**	1234	$C_{12}H_{25}O_4Si_2$	289
**Ser**	23	$C_{14}H_{34}ONSi_2$	288
**Ser**	12	$C_{14}H_{32}O_2NSi_2$	302
**Ser**	23	$C_{16}H_{40}O_2NSi_3$	362

**Ser**	123	$C_{17}H_{40}O_3NSi_3$	390
**Thr**	234	$C_{17}H_{42}O_2NSi_3$	376
**Thr**	1234	$C_{18}H_{42}O_3NSi_3$	404
**Tyr**	23456789	$C_{20}H_{38}ONSi_2$	364
**Val**	2345	$C_{12}H_{30}ONSi_2$	260
**Val**	12345	$C_{13}H_{30}O_2NSi_2$	288

a = “Carbons” indicates the carbons present in the derivative that is measured via GC-MS.

b = chemical formula of the derivative measured via GC-MS (derivatization process described in the method section)
